# SpaceBF: Spatial coexpression analysis using Bayesian Fused approaches in spatial omics datasets

**DOI:** 10.1101/2025.03.29.646124

**Published:** 2025-04-10

**Authors:** Souvik Seal, Brian Neelon

## Abstract

Advancements in spatial omics technologies have enabled the measurement of expression profiles of different molecules, such as genes (using spatial transcriptomics), and peptides, lipids, or N-glycans (using mass spectrometry imaging), across thousands of spatial locations within a tissue. While identifying molecules with spatially variable expression is a well-studied statistical problem, robust methodologies for detecting spatially varying co-expression between molecule pairs remain limited. To address this gap, we introduce a Bayesian fused modeling framework for estimating molecular co-expression at both local (location-specific) and global (tissue-wide) levels, offering a refined understanding of cell-cell communication (CCC) mediated through ligand-receptor and other molecular interactions. Through extensive simulations, we demonstrate that our approach, termed SpaceBF, achieves superior specificity and power compared to existing methods that predominantly rely on geospatial metrics such as bivariate Moran's I and Lee's L. Applying our framework to real spatial transcriptomics datasets, we uncover novel biological insights into CCC patterns across different cancer types.

## 1 Introduction

Technological advances in spatial omics [1, 2, 3] have enabled *in situ* profiling of varying molecules, including genes (via spatial transcriptomics (ST)) [4, 5, 6, 7], lipids or peptides (using mass spectrometry imaging (MSI)) [8, 9, 10, 11], and immune proteins (through multiplex immunofluorescence (mIF)) [12, 13, 14, 15], within tissues. The technologies offer distinct yet complementary biological insights, differing in their spatial resolution and the number of molecules they can detect (throughput). For example, next-generation sequencing (NGS)-based ST platform Visium (from 10X Genomics) [16] offers transcriptome-wide gene-expression profiling (throughput $\sim 20,000$) at a 55 $\mu m$ spot-level resolution. MALDI MSI-based platforms (from Bruker Daltonics [17] and others) offer profiling different types of molecules, such as peptides, lipids, nucleotides, proteins, metabolites, and N-glycans, (throughput $\sim 50-1000$) at 10 $\mu m$ spot-level resolution. The mIF platform PhenoCycler (from Akoya Biosciences) [18] enables protein profiling (throughput $\sim$ 40) at a 0.6 $\mu m$ cellular resolution. Despite these differences, the underlying data structure remains largely consistent across technologies and platforms, comprising a collection of spatial locations (from single or multiple samples) with observed expression or intensity of various molecules. Consequently, similar biostatistical questions arise, centering the spatial dynamics of molecules within the complex tissue or tumor microenvironment (TME) [19, 20, 21, 22, 23].

In the context of ST datasets, identifying spatially variable genes (SVGs), i.e., the genes exhibiting spatially structured expression patterns across the tissue, has gained significant attention [24, 25, 26, 27, 28, 29, 30, 31, 32, 33, 34, 35, 36, 37, 38]. It enables critical downstream analyses such as discovering potential biomarkers and defining tissue regions that influence cellular differentiation and function [39, 40, 41, 42]. Analogously, for mIF or imaging mass cytometry (IMC) datasets, innovative methods [43, 44, 45, 46, 47, 48, 49, 50, 51] have been proposed to understand the spatial distribution of immune cell types (defined by binarizing the expression profile of immune proteins) across the TME. Building upon this univariate framework, which typically analyzes one molecule at a time, another widely investigated problem has been spatial domain detection, i.e., deconvolving the tissue into distinct, spatially contiguous neighborhoods based on multivariate gene expression (ST) [52, 53, 54, 55, 56, 57, 58, 59, 60, 61, 62, 63] or immune cell type composition (mIF) [64, 65, 66, 67, 68, 69]. It aids mapping the molecular and functional landscape of tissues, elucidating disease progression, and guiding targeted therapies [70, 71, 72]. While some of the referenced methods can be adapted for use with MSI datasets, it is important to underscore the lack of sophisticated spatial functionalities of the existing bioinformatics toolboxes [73, 74, 75, 76].

While univariate and multivariate spatial analyses have garnered significant attention, a critical intermediate task remains underexplored: bivariate spatial co-expression analysis of molecule pairs at both the "local" (spot/cell-specific) and "global" (tissue-wide) levels. To emphasize the importance of accurately estimating pairwise spatial co-expression, we review cell-cell communication (CCC) [77, 78, 79, 80], through which cells exchange information via direct contact or signaling molecules (ligands) binding to other molecules (receptors) on the same or other cells. CCC regulates critical processes such as tissue development [81] and immune responses [82], and its disruption is linked to cancer [83]. Autocrine, juxtacrine, and paracrine signaling are three major pathways of CCC [84]. In autocrine signaling, ligands released by a cell bind to receptors on the same cell, while in juxtracrine and paracrine signaling, the ligands target adjacent and nearby cells. The study of ligand-receptor interactions (LRI), which involves identifying gene pairs (ligands and receptors) that show coordinated upregulation or downregulation across groups of cells, has become a fundamental approach for inferring CCC from single-cell RNA sequencing (scRNA-seq) datasets [85, 86, 87, 88, 89, 90, 91, 92, 93]. However, these approaches are prone to false positive interactions due to the lack of spatial context in scRNA-seq datasets, treating distant cell pairs similarly to nearby ones [94, 95, 96], which potentially leads to an overestimation of juxtracrine and paracrine signaling. ST datasets offer a natural avenue for improvement by enabling spatially constrained LRI analysis.

A limited number of tools exist for spatial LRI analysis or, more broadly, for assessing bivariate spatial co-expression of molecules in ST or MSI datasets. It should be emphasized that bivariate co-expression can manifest in two ways: (a) joint over- or under-expression within the same cells (correlation) and (b) joint over- or under-expression in neighboring cells (cross-correlation [97]). Some relevant methods include MERINGUE [98], Giotto [99], SpaGene [100], SpaTalk [101], SpatialDM [102], CellChat V2 [103], LIANA+ [104], and Copulacci [105]. We skip the approaches that jointly analyze multiple LR pairs

[106, 107]. Methods such as MERINGUE, Giotto, and SpaTalk provide only a global summary of spatial co-expression across a tissue, whereas others also offer local (spot/cell-specific) estimates. Let the standardized expression of two genes $(m, m')$ be $X^m(s)$ and $X^{m'}(s)$ at location $s$ for $s \in \{s_1, \ldots, s_n\}$, and $X^m = (X^m(s_1), \ldots, X^m(s_n))^T$, $X^{m'} = (X^{m'}(s_1), \ldots, X^{m'}(s_n))^T$. For a global summary of spatial co-expression, MERINGUE and SpatialDM leverage a popular geospatial metric termed the bivariate Moran's $I$ ($I_{BV}$) [108, 109], interpreted as the Pearson correlation between one variable and the spatial lagged version of the other [110, 111, 112]. Mathematically, $I_{BV} \propto (X^m)^T W X^{m'}$, where $W = [[w_{k_1k_2}]]$ is the spatial weight matrix that controls the spatial lagging. As $W$, MERINGUE uses a binary adjacency matrix based on the Delaunay triangulation [113] of the spatial locations ($w_{k_1k_2} = 1$ if locations $(s_{k_1}, s_{k_2})$ are connected, or 0 otherwise). SpatialDM uses a kernel covariance matrix or Gram matrix [114] based on the $L^2$ distance between locations ($w_{k_1k_2} = k_l(|s_{k_1} - s_{k_2}|^2)$, where $k_l$ is a kernel function with lengthscale parameter $l$ [115]). For local estimates of spatial correlation, SpatialDM considers the bivariate local Moran's $I$ ($I_{BV}^{local}(s)$) based on the local indicators of spatial association (LISA) approach [116]. The LIANA+ toolbox implements SpatialDM and introduces a comparable spatially weighted cosine similarity index. Of note, a newer package named Voyager [117] considers Lee's $L$ statistic [110] which has a slightly different formulation than $I_{BV}$. A critical yet often overlooked aspect of ST data analysis is that gene expression, measured in terms of unique molecular identifier (UMI) count, is inherently a discrete random variable (RV). However, the above methods assume normality upon a variance-stabilizing transformation [118, 119, 120], which may obscure true signals and have been widely criticized both within the ST literature [26, 55, 121, 122] and in broader contexts [123, 124, 125, 126]. Addressing this issue, Copulacci models a pair of genes as bivariate Poisson-distributed RVs, with their correlation in spatially adjacent cells captured using a Gaussian copula [127]. For inference, these methods typically rely on a permutation test [128].

Bivariate Moran's $I$ ($I_{BV}$) and Lee's $L$ statistics, implemented in MERINGUE, SpatialDM, LIANA+, and Voyager, are primarily recommended in spatial statistical literature as exploratory metrics for assessing cross-correlation rather than rigorous hypothesis testing tools [129, 97]. We have noticed that even when two variables are independently simulated with certain spatial covariance structures, the unmodeled spatial autocorrelation introduces a confounding effect on the bivariate association, leading to significantly inflated type 1 error rates (see simulation studies). A similar issue is well documented, as extensive literature highlights the limitations of using simple Pearson's correlation to assess dependencies between two variables in the presence of spatial autocorrelation [130, 131, 132, 133, 134]. By extension, since $I_{BV}$ and Lee's $L$ are fundamentally Pearson's correlation applied to spatially lagged variables, they may also be prone to identical pitfalls. Furthermore, these spatially weighted association indices, being model-free, are not able to seamlessly adjust for cell-level covariates such as cell type, a limitation also present with Copulacci. As a side note, mapping to the aforementioned CCC pathways, simple Pearson's correlation between ligand and receptor can be interpreted as a proxy for autocrine signaling, while cross-correlation may reflect a combination of juxtacrine and paracrine signaling.

We approach the bivariate spatial co-expression detection as a generalized linear regression problem, modeling a molecule $m$ as the outcome and the other molecule $m'$ as the predictor (Eqs. 1 and 2). For ST datasets, gene expression or UMI count is modeled as a negative binomial (NB)-distributed RV, while an alternative Gaussian model is considered for continuous cases. The regression coefficients, both intercept ($\beta_0^{mm'}(s)$) and slope ($\beta_1^{mm'}(s)$), are assumed to vary across locations ($s$) exhibiting spatial dependency. Known as the spatially varying coefficients (SVC) model [135], this framework provides exceptional flexibility and precision in capturing locally changing co-expression patterns through $\beta_1^{mm'}(s)$. A large positive $\beta_1^{mm'}(s)$ suggests strong positive co-expression at location $s$, i.e., joint up or down-regulation, whereas a large negative value indicates avoidance or repulsion. The average of $\beta_1^{mm'}(s)$'s, $\overline{\beta_1^{mm'}} = \sum_k \beta_1^{mm'}(s_k)/n$, provides a summary of the global co-expression pattern. Similar models have been widely used in fields such as disease mapping [136, 137], econometrics [138, 139], ecological studies [140, 141], and neuroimaging research [142, 143]. In the Bayesian paradigm, the spatial dependency of $\beta_0^{mm'}(s)$'s and $\beta_1^{mm'}(s)$'s, is typically modeled using a conditionally autoregressive (CAR) [144, 145, 146] or Gaussian process (GP) priors [147, 97, 148]. In contrast, we introduce a locally adaptive spatial Gaussian Markov random field (GMRF) prior [149] based on the concepts of fusion penalties [150, 151, 152] and horseshoe prior [153, 154, 155], extending a related work in the frequentist setup [156]. Briefly, the prior incorporates the spatial similarity between two adjacent locations, $(s_{k_1}, s_{k_2})$, by encouraging $|\beta_0^{mm'}(s_{k_1}) - \beta_0^{mm'}(s_{k_2})| \approx 0$ and $|\beta_1^{mm'}(s_{k_1}) - \beta_1^{mm'}(s_{k_2})| \approx 0$. Adjacency is defined using the minimum spanning tree (MST) [157, 158, 159] constructed with the $L^2$ distances between locations as edge weights. The MST of a connected, weighted graph offers the most economical connectivity between all vertices without cycles, minimizing the total edge weight. A detailed discussion on the choice of MST as the underlying spatial graph, along with its implications for the resulting GMRF prior, is provided later. The proposed method, SpaceBF, is evaluated against existing approaches using realistic simulation scenarios, showcasing its high specificity and power. It is further applied to two ST datasets: one on cutaneous melanoma for spatial LRI analysis and another on cutaneous squamous cell carcinoma for keratin-interaction analysis.

## 2 Result

### 2.1 Real data analysis

#### 2.1.1 Melanoma ST dataset

We analyzed a cutaneous melanoma dataset [39] on a long-term survivor (10+ years) collected using the ST technology [4], comprising 293 spots, each 100 $\mu m$ in size and at 200 $\mu m$ center-to-center distance. There are 16,148 genes, forming 1,180 known ligand-receptor (LR) pairs as available from CellChatDB [88]. There are three major pathologist-annotated regions as seen in the histology image (Fig. 1A), collected from Thrane et al. (2018), and 6 major cell types (Fig. 1B) predicted using the RCTD [160] package based on overall gene expression [102]. After filtering out genes with extremely low expression ($< 0.2 \times 293 \approx 59$ reads), 161 LR pairs remain, which are examined using our method SpaceBF. SpaceBF first constructs a minimum spanning tree (MST) based on the spatial coordinates of the spots (Fig. 1C). Then, for every LR pair: $(m', m)$, it considers Eq. 2 with the receptor expression as $X^m(s_k)$ and the ligand expression as $X^{m'}(s_k)$, with $s_k$ representing a spot. Using the global test, SpaceBF identified 53 LR pairs at level 0.05 (33 at FDR $< 0.1$). The estimated slope surface $\beta_1^{mm'}(s_k)$ of different LR pairs exhibits distinct patterns. To highlight these differences, we classify the detected LR pairs into 3 major patterns (Fig. 1E) based on hierarchical clustering [161] of the standardized vector $\boldsymbol{\beta}_1^{mm'*} = (\beta_1^{mm'}(s_1) - \overline{\beta_1^{mm'}}, \ldots, \beta_1^{mm'}(s_n) - \overline{\beta_1^{mm'}})^T / \sigma_\beta^{mm'}$, where $\overline{\beta_1^{mm'}} = \sum_k \beta_1^{mm'}(s_k)/n$ and $\sigma_\beta^{mm'}$ are the tissue-wide average and the SD of estimated $\beta_1^{mm'}(s_k)$'s, respectively. 20 LR pairs follow pattern 1, while 22 and 11 LR pairs correspond to patterns 2 and 3, respectively. Similarly, the spots are grouped into 4 clusters based on the spot-level vectors of slopes corresponding to the 53 detected LR pairs (Fig. 1D). It is visibly clear that clusters 1 and 3 correspond to the melanoma region, while clusters 2 and 4 loosely correspond to the stroma and lymphoid regions, respectively. Returning to the LR patterns, in Fig. 1F, the LR pairs are arranged sequentially from pattern 1 to 3, highlighting the enrichment of their interaction in three major cell types. For example, $\sum_{k \in \mathrm{B/T\ cells}} \beta_1^{mm'*}(s_k)$ represents the enrichment within B/T cells relative to the average enrichment $\overline{\beta_1^{mm'}}$ and scaled by the SD. The levels "highest," "medium," and "lowest" indicate the degree of enrichment, with "highest" corresponding to the greatest or most positive enrichment and so on. The majority of LR pairs following pattern 1 exhibit higher or more positive interaction in B/T cells within the lymphoid region (some in CAF cells) and more negative interaction (avoidance or repulsion) in the melanoma region or cells. Pattern 2 mostly corresponds to LR pairs with the highest enrichment in CAF cells, while pattern 3 clearly corresponds to the pairs with the highest enrichment in melanoma cells. Next, we closely examine a few LR pairs to gain deeper insight into the biological significance of their estimated slope surfaces. The LR pair (IGF2, IGF1R) [162] corresponds to pattern 1 and demonstrates a negative association overall, with an estimated average slope of $\overline{\beta_1^{mm'}} = -0.212$, and the $p$-value $= 0.024$, which is consistent with a visual inspection (Fig. 1G). It could indicate a lack of binding between these genes, which would be a generally favorable factor for the survivor [163]. Setting the insignificant $\beta_1^{mm'*}(s_k)$ values to 0 based on the local test, the negative interaction found in the melanoma region has the highest credibility. The pair (PTPRC, CD22) [164] follows pattern 2, with $\overline{\beta_1^{mm'}} = 0.422$ and $p$-value of $6.28 \times 10^{-6}$. PTPRC, also known as CD45, is a facilitator of T-cell receptor (TCR) and B-cell receptor (BCR) signaling [165], while CD22 is primarily an inhibitor of BCR signaling [166]. Their overall positive co-expression, particularly in the lymphoid region, is likely associated with a balanced B cell regulation, helping to prevent autoimmunity and promoting lymphoid growth in other regions as part of the immune response. The final LR pair we discuss is (SPP1,

CD44) [167], which follows pattern 3, exhibiting a highly positive overall co-expression with $\overline{\beta_1^{mm'}} = 0.79$ and $p$-value of $1.03 \times 10^{-6}$. This strong interaction displays a decreasing gradient from the melanoma region to the lymphoid region, which aligns with its known role in dysregulated cytoskeletal remodeling [168], facilitating melanoma cell invasion into surrounding tissues. Further discussion is provided in the Supplementary Material.

#### 2.1.2 cSCC ST dataset

We analyzed a cutaneous squamous cell carcinoma (cSCC) dataset [169] on a patient sample with a histopathologic subtype of "moderately differentiated" cSCC [170]. The dataset was collected using the ST technology with 621 spots, each of size 110 $\mu m$ and a center-to-center distance of 150 $\mu m$. There are $16,643$ genes of which 45 are keratins (14 after filtering low-count genes, $< 0.2 \times 621 \approx 124$ reads). These keratins can be classified into two types: 1) type 1, which includes KRT10, KRT14–KRT17, and KRT23, and 2) type 2, which includes KRT1, KRT2, KRT5, KRT6A, KRT6B, KRT6C, KRT78, and KRT80. The keratins pair together to form intermediate filaments, providing structural support to epithelial cells [171]. In the context of cSCC and other carcinomas, keratins are emerging as highly significant targets for therapeutic intervention [172, 173, 174]. Of note, some of the keratins belong to the GO term: "keratinocyte differentiation" (GO:0030216) and were reported to exhibit strong spatial correlation in an earlier work [175] involving the same dataset. We utilized SpaceBF to investigate the binding between type 1 and type 2 keratins, resulting in a set of 48 keratin pairs. In the histology image (2A), the deep blue areas at the top and left sides correspond to tumor regions, while the whitish region at the bottom represents a non-tumor region, possibly composed of keratinized layers and stroma [176]. However, the tumor and non-tumor regions are not clearly delineated, a feature characteristic of moderately differentiated cSCC, with the spatial clusters obtained using the BayesSpace package [52] on the transcriptome-wide gene expression profile (2C) partially elucidating this distinction. The constructed MST is shown in 2B. Using the global test, SpaceBF identified 39 keratin pairs at a significance level of 0.05 (41 at an FDR $< 0.1$), suggesting that most pairs bind to each other, albeit to varying degrees. Similar to the earlier analysis,

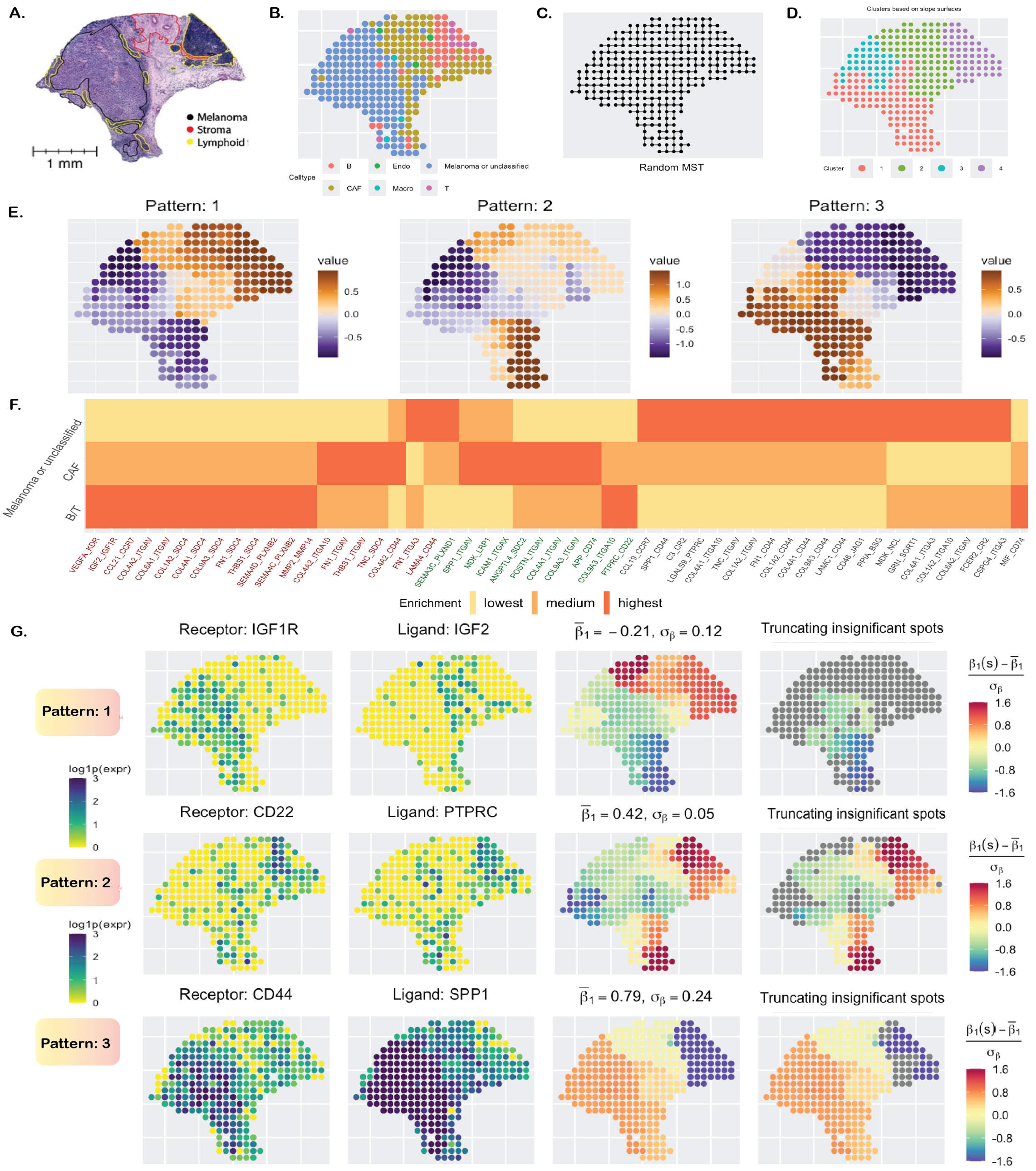

Figure 1: Cutaneous melanoma data analysis. **A**. Annotated H&E-stained image. **B**. Cell types based on gene expression. **C**. Minimum spanning tree (MST) capturing the spatial structure. **D**. Clustering of spots based on centered and scaled estimates of slope surfaces of 53 statistically significant LR pairs. **E**. Three main spatial patterns of the estimated surfaces. **F**. Enrichment of LR interactions in three major cell types, with LR names arranged and color-coded according to their respective patterns. **G**. The first two columns show the expression of three LR pairs. The third column displays the centered and scaled slope surfaces. In the fourth column, insignificant spot-level slope estimates are greyed.

we classify the detected slope surfaces into 3 major patterns (Fig. 2D) based on hierarchical clustering of the standardized vector $\boldsymbol{\beta}_1^{mm'*}$. We represent the keratin pairs as bipartite graphs between type 1 and 2 keratins under each pattern (Fig. 2E). One important observation is that type 2 keratins KRT6A, KRT6B, and KRT6C are isoforms of keratin 6 [177] and thus, co-express highly, meaning their binding patterns with any specific type 1 keratin should be similar, as correctly identified by SpaceBF. For instance, the slope surfaces of KRT10 with KRT6A, 6B, and 6C all align with pattern 1, while the slope surfaces of KRT16 with KRT6A, 6B, and 6C all correspond to pattern 2. This consistency underscores the reliability of SpaceBF in identifying true local patterns. In Fig. 2F, we present the estimated slopes for KRT17, which is a well-established therapeutic target in various cancers [178, 179, 180], binding with three type 2 keratins: KRT80 (pattern 1, $p$-value $= 0.006$), KRT78 (pattern 2, $p$-value $= 0.03$), and KRT6B (pattern 3, $p$-value $= 5.59 \times 10^{-6}$). Notably, the average slope estimates for KRT17-KRT80 and KRT17-KRT78 interactions are small ($\approx 0.1$), whereas for KRT17-KRT6B, the average slope is substantially higher at 0.82, with the highest local estimates observed mostly in tumor regions. These trends are also evident from the individual expression profiles provided in Fig. 2F. Although the expression patterns of KRT80 and KRT78 appear similar, a closer examination reveals that KRT80 exhibits a thicker band of expression on the left, specifically within the tumor regions. This distinction contributes to the difference in co-expression patterns of KRT17-KRT80 and KRT17-KRT78. As previously noted, both association levels are low, also indicated by the small number of significant spots identified by the local test, 72 and 49, respectively.

### 2.2 Simulation studies

In simulation design 1, two NB-distributed random variables (RVs), $(\mathbf{X}^m, \mathbf{X}^{m'})$ are generated based on the true model from Eq. 2. The spatial autocorrelation of the variables is simulated using a Gaussian process (GP) model [97] with a spatial covariance matrix $H$ based on an exponential kernel and the $L^2$ distance. The locations are considered to be the same as the cutaneous melanoma dataset, i.e., $n = 293$ (more details on the design provided in the Methods section). From Fig. 3A, we notice how the structure of $H$ changes as the lengthscale $l$ varies. The off-diagonal elements of $H$ $(H_{k_1k_2})$ can range between 0

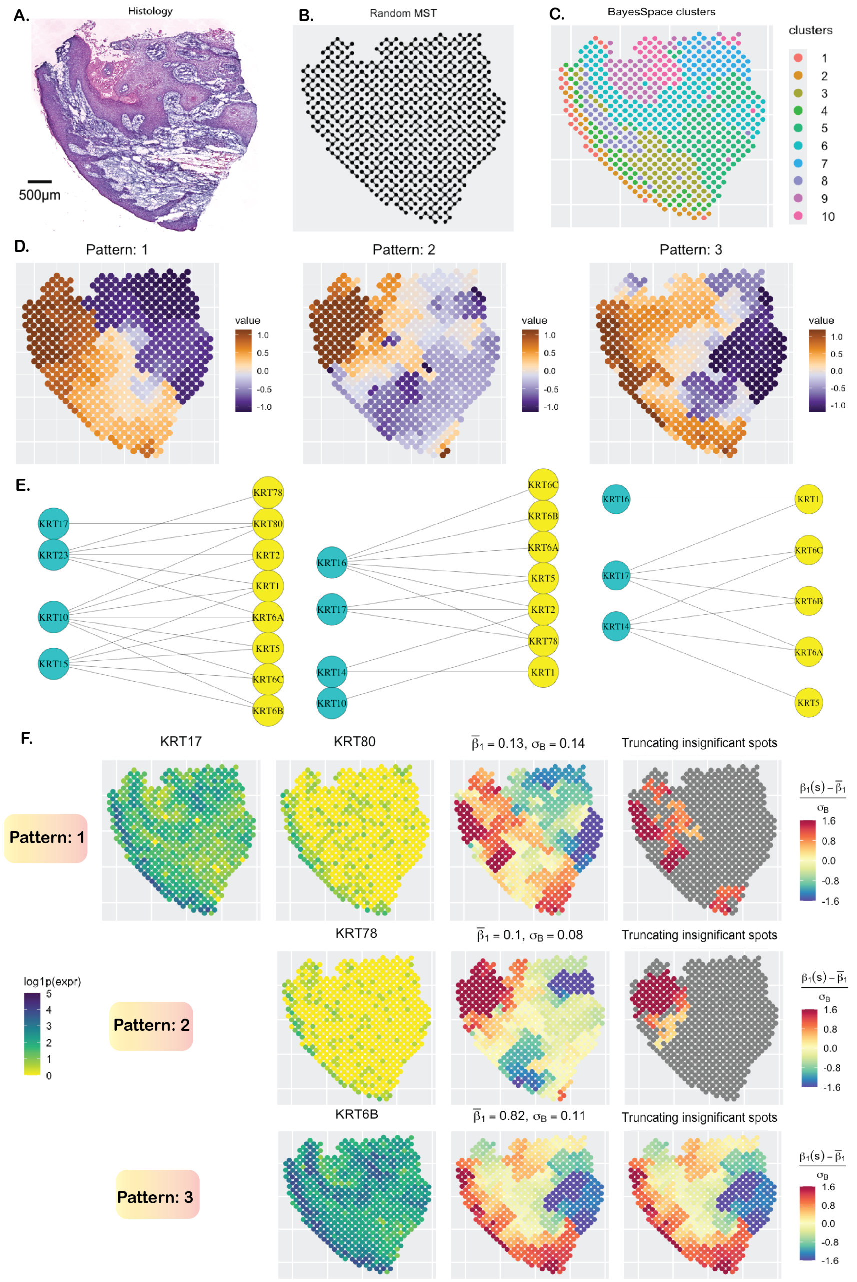

Figure 2: Cutaneous squamous cell carcinoma data analysis. **A**. H&E-stained image. **B**. MST capturing the spatial structure. **C**. Spatial clusters obtained using BayesSpace package. **D**. Three main spatial patterns of the estimated surfaces. **E**. Bipartite graphs between type 1 and type 2 keratins based on the spatial pattern they follow. **F**. Study of the interaction between the type 1 keratin KRT17 and three distinct type 2 keratins, each exhibiting a unique estimated slope surface pattern. The insignificant spot-level slope estimates are greyed in the last column.

and 1. When $l = 0.6$, only the nearest locations $(k_1, k_2)$ exhibit high $H_{k_1k_2}$ with most of the other values being close to 0. In contrast, for $l = 18$, the majority of location pairs have high $H_{k_1k_2}$ ($\approx 1$), inducing an exceptionally strong spatial autocorrelation in both variables. As described later, in the simulation design 1, $\mathbf{X}^{m'}$ is first simulated, followed by $\mathbf{X}^m$, which is simulated based on Eq. 2 with a constant slope, $\beta_1^{mm'}(s) = \nu$. To visibly understand how $\nu$ might affect the relationship between $\mathbf{X}^m$ and $\mathbf{X}^{m'}$, in Fig. 3B, we show the spatial expression of $\mathbf{X}^m$ for the same $\mathbf{X}^{m'}$ but three different values of $\nu$, $\{-0.75, 0, 0.75\}$. It is somewhat evident that nonzero $\nu$'s result in a visibly positive or negative association, while $\nu = 0$ produces a random pattern of $\mathbf{X}^m$. In Fig. 3C, we show the type 1 error ($\nu = 0$) and power ($\nu \neq 0$) comparison of the different methods, including SpaceBF, for three values of the lengthscale $l$. When $l = 3.6$, both variables exhibit considerable spatial autocorrelation, yet SpaceBF maintains the correct type 1 error. In contrast, all other methods suffer from inflated type 1 errors. Notably, simple Pearson's correlation, while still inflated, performs better in controlling type 1 error compared to methods based on bivariate Moran's $I$ or Lee's $L$. Although SpaGene does not rely on these traditional metrics, it also fails to control type 1 error effectively. The issue becomes more pronounced with increasing $l$, indicating a higher degree of spatial autocorrelation. Notably, Lee's $L$ exhibits the highest inflation in the majority of cases. SpaceBF also retains the high detection power throughout all three cases. Although the power declines slightly, as expected, due to a decrease in effective sample size from increased spatial autocorrelation. In summary, the simulation effectively demonstrates the specificity and power of our method.

In simulation design 2, we generate $(\mathbf{X}^m, \mathbf{X}^{m'})$ jointly as bivariate spatially correlated NB-distributed RVs. This setup is more complex than the previous one, as the association is non-linear and driven by the Kronecker product-based spatial covariance structure (see the Methods section). The methods, except SpaceBF, perform poorly in terms of the type 1 error for $l \geq 1.8$. When the spatial autocorrelation is the weakest ($l = 0.6$), Pearson's correlation performs well as expected, while spatially weighted indices still show a slight inflation. SpaceBF achieves controlled type 1 error and steady detection power across varying $l$'s. As earlier, the power decreases as the effective sample size decreases. Together, these two simulation designs demonstrate SpaceBF's robustness under complex data generation processes. Finally, we argue

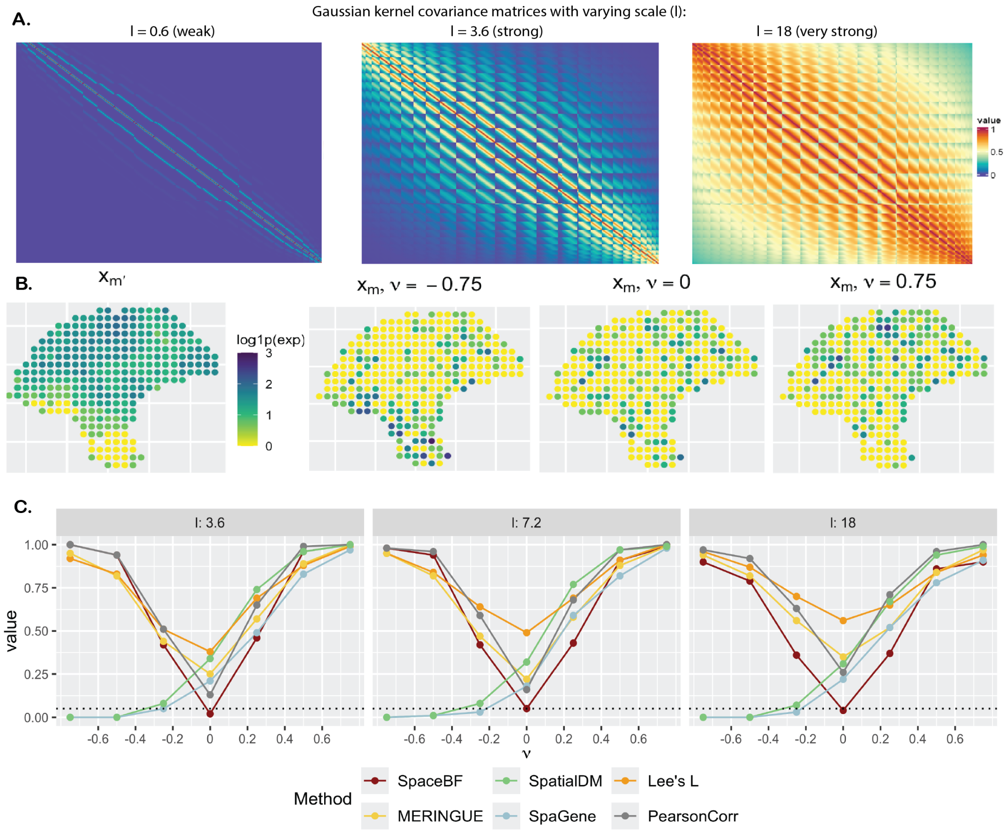

Figure 3: Comparison of global tests under the simulation design 1. **A**. Heatmap of the spatial covariance matrix $H$ with an exponential kernel and $L^2$ distance, for varying values of the lengthscale parameter $l$. **B**. Simulated $\mathbf{X}^m$ based on Eq. 2, for a fixed $\mathbf{X}^{m'}$ but different values of the constant slope $\beta_1^{mm'}(s) = \nu$. **C**. Performance of the methods in terms of type 1 error ($\nu = 0$) and power ($\nu \neq 0$). The dotted line represents the significance level 0.05.

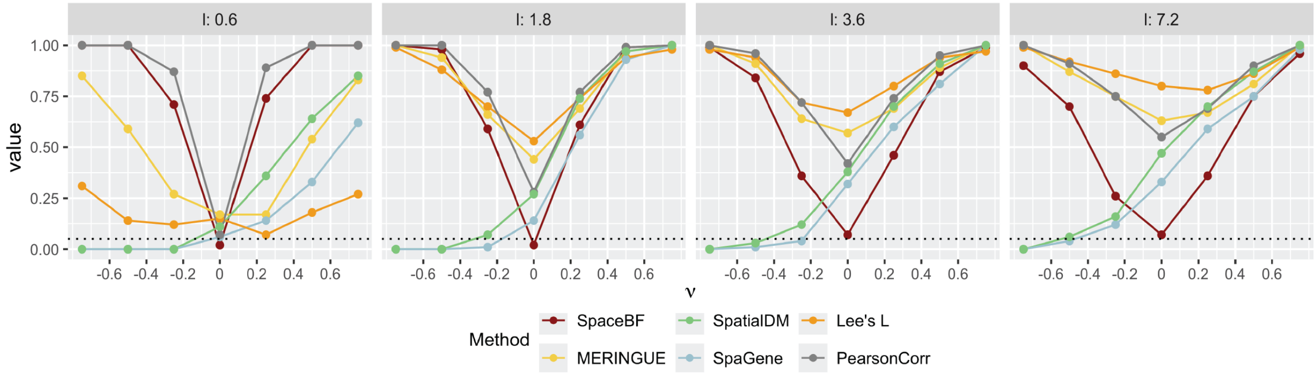

Figure 4: Comparison of global tests under simulation design 2 for lengthscale $l$ between $\{0.6, 1.8, 3.6, 7.2\}$.

that incorporating $\beta_0^{mm'}(s)$ in SpaceBF accounts for the spatial autocorrelation of variable $m$, thereby mitigating bias in the association analysis of $(m, m')$. As previously noted, Pearson's correlation is already recognized as suboptimal in such scenarios, and spatially weighted indices, essentially Pearson's correlation between spatially lagged variables, remain susceptible to spurious detections. Recall that MERINGUE uses the Delaunay triangulation-based network with binary spatial weight matrix $W$, while SpatialDM uses a continuous spatial weight matrix having a similar form as $H$ for a particular choice of the lengthscale. Lee's $L$ is based on a binary $k-$NN network in our study. Hence, the performance of these methods could be sensitive to choices of the spatial weight matrices, i.e., different networks or lengthscale $l$ values. SpatialDM and SpaGene focus solely on the joint over-expression of molecules, neglecting joint under-expression. As a result, for most values of $\nu < 0$, these methods show almost no detection power.

### 3 Discussion

We have developed a rigorous framework for studying spatial co-expression of a pair of molecules in the context of spatial transcriptomics (ST) and mass spectrometry imaging (MSI) datasets. Existing tools mostly rely on two exploratory geospatial metrics, namely, bivariate Moran's $I$ [108] and Lee's $L$ [110], which lead to highly spurious association inference as demonstrated by our simulation studies. Our proposed approach, SpaceBF, builds on the widely used spatially varying coefficients model [135], effectively capturing spatial autocorrelation and locally varying co-expression patterns. We introduce a novel spatial GMRF prior based on the minimum spanning tree (MST) network [181, 159] between locations and elucidate its theoretical properties, inspired by the concepts of fused lasso [152] and horseshoe prior [155], drawing motivation from a recent frequentist study in a different domain [156]. By integrating this prior directly within a complex negative binomial regression framework [182], our method is broadly applicable across various analytical contexts. We perform a rigorous evaluation of the method in challenging simulation scenarios and apply it to two ST datasets, demonstrating its strong performance in detecting cell-cell communication through ligand-receptor and other molecular interactions.

Utilizing the MST as the underlying spatial graph provides several advantages, including (1) its uniqueness, eliminating the need to tune additional hyperparameters such as the lengthscale in Gaussian process regression [97], and (2) reduced computational complexity by inducing a sparser precision matrix. In our simulations, where the spatial dependency is always simulated using a kernel covariance matrix with varying lengthscales, our MST-based approach has performed exceptionally well, highlighting its robustness. However, fixing the spatial structure with a spanning tree may not always be optimal, as it could omit certain important edges [183]. In future work, we will explore the possibility of treating the spanning tree as a so-called unknown parameter and update it iteratively [184]. While adjusting for covariates in our model is straightforward, it would be valuable to investigate this further, particularly to better understand the cell type-specific properties of co-expression in different real data applications. Additionally, our model might be extended to incorporate multiple predictors with spatially varying slopes, a direction we plan to pursue in the future with careful attention to computational feasibility and multicollinearity of the predictors.

## 4 Methods

### 4.1 Gaussian and NB linear model

We assume a single sample or image with $n$ spots/cells. Let $X^m(s_k)$ and $X^{m'}(s_k)$ denote the expression of a pair of molecules $m$ and $m'$, and $Z(s_k)$ be a vector of $p$ covariates observed at spot/cell location $s_k$, for $k \in \{1, \dots, n\}$. For example, $Z(s_k)$ can be the cell type indicator or a vector of cell type proportions [160]. We consider the following spatially varying coefficients (SVC) model [135]

$$X^m(s_k) = \beta_0^{mm'}(s_k) + X^{m'}(s_k)\beta_1^{mm'}(s_k) + Z(s_k)^T\alpha_m + \epsilon(s_k), \;\; k = 1, \dots, n, \tag{1}$$

where $\beta_0^{mm'}(s_k)$ and $\beta_1^{mm'}(s_k)$ denote spatially varying intercept and slope, respectively, $\alpha_m$ is a fixed effect vector, and $\epsilon(s_k)$ is an independent error term. To interpret the model, a significantly positive $\beta_1^{mm'}(s_k)$ implies that the molecules $(m, m')$ co-express at the location $s_k$, while a significantly negative value suggests avoidance. Intuitively, $\beta_0^{mm'}(s_k)$ accounts for spatial autocorrelation of molecule $m$. More discussion on the underlying bivariate spatial process is provided in the Supplementary Material. For a count-valued $X^m(s_k)$ (e.g., genes in the ST datasets), we consider a spatially varying negative binomial

(NB) distribution [182] as $NB(\psi_m(s_k), r_m)$ with the failure probability $\psi_m(s_k)$ modeled in the following way

$$\begin{aligned} \eta_m(s_k) &= \beta_0^{mm'}(s_k) + X^{m'}(s_k)\beta_1^{mm'}(s_k) + Z(s_k)^T\alpha_m \\ p(X^m(s_k)|\psi_m(s_k), r_m) &\propto (1-\psi_m(s_k))^{r_m}\psi_m(s_k)^{X^m(s_k)}, \quad \psi_m(s_k) = \frac{\exp(\eta_m(s_k))}{1+\exp(\eta_m(s_k))}, \end{aligned} \tag{2}$$

where $p(.|...)$ denotes the conditional probability mass function (PMF) and the dispersion parameter $r_m$ is assumed to be constant across locations. Admittedly, this model is limited as the count-valued nature of the molecule $X^{m'}(s_k)$ is not prioritized, appearing as a spatially varying predictor. Modeling $(X^m(s_k), X^{m'}(s_k))$ jointly as bivariate NB (BNB) distributed RVs is a possibility that we do not pursue as there are varying definitions of a BNB distribution (other than via Copula) [185, 186, 187, 188], leading to restrictive correlation structures and inefficient MCMC samplers.

The models in Eqs. 1 and 2 are over-parametrized and do not incorporate spatial dependency between $\beta_0^{mm'}(s_k)$'s and $\beta_1^{mm'}(s_k)$'s, which we discuss next. Let $G = (V, E)$ denote the MST between the locations constructed using the $L^2$ distance: $|s_{k_1} - s_{k_2}|^2$ for a pair $(s_{k_1}, s_{k_2})$, where $V$ and $E$ are the sets of vertices and edges, respectively. Given a connected, weighted graph, the MST is an acyclic subgraph that connects all vertices and minimizes the sum of the weights of the included edges. Because of this property, MST is routinely used to develop transportation and telecommunication networks [189]. For a regular grid, MST is not unique as the inter-point distances are not distinct. However, a unique random MST can be curated by simply adding small random values to the distances [181, 159].

### 4.2 Spatial modeling

#### 4.2.1 Spatial fused lasso

In a recent study [156] of the temperature-salinity relationship in the Atlantic Ocean, Li et al. (2019) consider Eq. 1 and elegantly promote spatial homogeneity of the coefficients by considering fused lasso penalties [150, 190]: $|\beta_0^{mm'}(s_{k_1}) - \beta_0^{mm'}(s_{k_2})| \approx 0$ and $|\beta_1^{mm'}(s_{k_1}) - \beta_1^{mm'}(s_{k_2})| \approx 0$ for $(s_{k_1}, s_{k_2}) \in E$, in a frequentist setup. These constraints are intuitive, as it is reasonable to expect both the degree of co-expression, $\beta_1^{mm'}(s)$, and the effect of "unmeasured" factors, $\beta_0^{mm'}(s)$, to remain homogeneous across adjacent or connected locations. Extending this idea, a Bayesian fused lasso [191, 152] approach can be considered with Laplacian priors on the pair-wise differences of the coefficients as

$$\begin{aligned}
\pi(\boldsymbol{\beta}_0^{mm'}|\ldots) &\propto \prod_{(s_{k_1},s_{k_2})\in E} \exp\left(-\frac{\lambda_0}{\sigma}|\beta_0^{mm'}(s_{k_1}) - \beta_0^{mm'}(s_{k_2})|\right), \ \boldsymbol{\beta}_0^{mm'} = (\beta_0^{mm'}(s_1), \ldots, \beta_0^{mm'}(s_n))^T, \\
\pi(\boldsymbol{\beta}_1^{mm'}|\ldots) &\propto \prod_{(s_{k_1},s_{k_2})\in E} \exp\left(-\frac{\lambda_1}{\sigma}|\beta_1^{mm'}(s_{k_1}) - \beta_1^{mm'}(s_{k_2})|\right), \ \boldsymbol{\beta}_1^{mm'} = (\beta_1^{mm'}(s_1), \ldots, \beta_1^{mm'}(s_n))^T,
\end{aligned} \tag{3}$$

where $\sigma^2$ is the variance of the error term $\epsilon(s_k)$, $\lambda_0$ and $\lambda_1$ are regularization parameters that control the strength of fusion and usually follow gamma priors. Scaling by $\sigma$ inside the exponents in Eq. 3 is only done in the case of Eq. 1, and could be omitted, particularly when $\mathbf{X}^m$ is scaled. We discuss the resemblance of the prior to the intrinsic CAR (ICAR) prior [145] and more generally, the intrinsic GMRF (IGMRF) prior [149] in the Supplementary Material. Theoretically, using the $L_1$ distance seems appealing, as it has the potential to achieve better spatial smoothing by "exactly" fusing coefficient values at adjacent locations, unlike the $L_2$ distance implied by the ICAR prior. This is analogous to how lasso regression enforces sparsity in solutions, while ridge regression only shrinks effect sizes toward 0 [192]. For transparency, such a spatial fused lasso prior has already been proposed in the existing literature [184, 193].

#### 4.2.2 Spatial fused horseshoe

In variable selection problems, failure of the Bayesian lasso or Laplacian prior to achieve exact sparsity, unlike the frequentist analog, has been reported, while also underestimating larger effect sizes [194, 195, 196]. Consequently, the Bayesian fused lasso might struggle to promote spatial contiguity and preserve distinct local features simultaneously. For variable selection, the advantages of the horseshoe prior have been convincingly demonstrated to handle unknown sparsity and large outlying signals [153, 197, 198]. The horseshoe prior belongs to the class of global-local shrinkage priors [199], characterized by a "global" hyperparameter that controls overall shrinkage, while "local" hyperparameters control shrinkage per coefficient. Following recent developments on Bayesian fused horseshoe approach [155, 200], we assume that

$$\begin{aligned}
\beta_0^{mm'}(s_{k_i^1}) - \beta_0^{mm'}(s_{k_i^2})|\Lambda_{0i}^2, \tau_0^2, \sigma^2 &\sim N(0, \Lambda_{0i}^2\tau_0^2\sigma^2), \ \ \Lambda_{0i} \sim C^+(0,1), \ \ \tau_0 \sim C^+(0,1) \\
\beta_1^{mm'}(s_{k_i^1}) - \beta_1^{mm'}(s_{k_i^2})|\Lambda_{1i}^2, \tau_1^2, \sigma^2 &\sim N(0, \Lambda_{1i}^2\tau_1^2\sigma^2), \ \ \Lambda_{1i} \sim C^+(0,1), \ \ \tau_1 \sim C^+(0,1)
\end{aligned} \tag{4}$$

independently, where $(s_{k_i^1}, s_{k_i^2})$ are the nodes or locations associated with the $i$-th edge from $E$. $\tau_0$ and $\tau_1$ are the global hyper-parameters, $\Lambda_{0i}$'s and $\Lambda_{1i}$'s are the local hyperparameters, and $C^+$ stands for the half-Cauchy distribution. The resulting conditional prior PDFs of $(\boldsymbol{\beta}_0^{mm'}, \boldsymbol{\beta}_1^{mm'})$ are

$$\begin{aligned} \pi(\boldsymbol{\beta}_0^{mm'}|\ldots) &\propto \prod_{i=1}^{p} \frac{1}{\Lambda_{0i}\tau_0\sigma} \exp\left[-\frac{(\beta_0^{mm'}(s_{k_i^1}) - \beta_0^{mm'}(s_{k_i^2}))^2}{2\Lambda_{0i}^2\tau_0^2\sigma^2}\right] \\ \pi(\boldsymbol{\beta}_1^{mm'}|\ldots) &\propto \prod_{i=1}^{p} \frac{1}{\Lambda_{1i}\tau_1\sigma} \exp\left[-\frac{(\beta_1^{mm'}(s_{k_i^1}) - \beta_1^{mm'}(s_{k_i^2}))^2}{2\Lambda_{1i}^2\tau_1^2\sigma^2}\right] \end{aligned} \tag{5}$$

The spatial horseshoe prior, with its half-Cauchy priors on both global and local hyperparameters, has heavy tails that allow large differences between neighboring locations to escape excessive shrinkage, unlike the Laplacian prior. At the same time, its infinitely tall spike at zero strongly shrinks small differences toward zero. This dual behavior enables it to effectively preserve spatial contiguity while still capturing sharp local variations. The relation of such a locally adaptive fusion prior to the general class of GMRF priors was first highlighted by Faulkner and Mining (2017) [201] in a longitudinal context, encouraging fusion between coefficients across time points. We provide further discussion on this relation in the Supplementary Material. We use the Polya-Gamma data augmentation strategy [202] for developing the Gibbs sampling algorithm for the NB regression (Eq. 2), as detailed in the Supplementary Material.

One critical aspect that deserves elucidation is the assumption of independence between edge-wise differences. Specifically, for any two edges $i, i'$, $\beta_1^{mm'}(s_{k_i^1}) - \beta_1^{mm'}(s_{k_i^2})$ and $\beta_1^{mm'}(s_{k_{i'}^1}) - \beta_1^{mm'}(s_{k_{i'}^2})$ are assumed to be independent in Eq. 4, conditional on the hyperparameters. To see how this assumption could be problematic for a general graph (not the MST), we briefly highlight one example from Rue and Held (2005) [149] provided in the context of IGMRF priors. Suppose there are only three locations A, B, and C, all neighbors of each other. Letting $e_1 = \beta_1^{mm'}(A) - \beta_1^{mm'}(B)$, $e_2 = \beta_1^{mm'}(B) - \beta_1^{mm'}(C)$, and $e_3 = \beta_1^{mm'}(C) - \beta_1^{mm'}(A)$, Eq. 4 proceeds to assume $e_1, e_2, e_3$ are independent and normally distributed with non-identical parameters. However, we notice that there is a "hidden" linear constraint, $e_1 + e_2 + e_3 = 0$, that directly contradicts the model assumption of independence. Analogously, using a highly connected or dense spatial neighborhood graph $G$ introduces numerous hidden constraints corresponding to the cycles in $G$. Interestingly enough, as demonstrated in Theorem 1 of the Supplementary Material, the hidden constraints do not require to be explicitly accounted for since the posterior sampling distribution of $\boldsymbol{\beta}_0$ and $\boldsymbol{\beta}_1$ (based on the priors in Eq. 5) remains unaffected. However, penalizing too many edge-wise differences and the implicit dependency might lead to over-smoothing and loss of local structures. Thus, the use of MST becomes crucial, as it is acyclic (no hidden constraints) and eliminates redundant relationships. Additionally, a sparser $G$ enables faster Cholesky decomposition of the precision matrix, enhancing computational efficiency.

#### 4.3 Hypothesis testing

We consider two types of hypothesis tests: 1) global test: to determine the significance of overall association across the entire tissue domain, based on the credible interval [203] of the average slope $\overline{\beta_1^{mm'}} = \frac{1}{n}\sum_{k=1}^{n} \beta_1^{mm'}(s_k)$, and 2) local test: to determine the significance of location-level association directly based on the credible interval of $\beta_1^{mm'}(s_k)$. In the genomic context, having a measure analogous to the frequentist $p$-value is often beneficial. To address this, we utilize the probability of direction ($p_d$), as implemented in the $R$ package bayestestR [204, 205], which closely resembles a frequentist $p$-value as $p_{\text{two-sided}} = 2(1 - p_d)$.

#### 4.4 Simulation design

We consider two different simulation designs as outlined below, for assessing the type 1 error and power of the model proposed in Eq. 2. The locations, on which the variables are simulated, are the same as the previously discussed cutaneous melanoma dataset ($n = 293$). We have observed that the results remain unaffected when a randomly generated set of locations or other real data-based sets of locations are used.

##### 4.4.1 Simulation design 1

In the first design, we directly consider the model from Eq. 2 to generate $(\mathbf{X}^m, \mathbf{X}^{m'})$ based on two steps. First, we generate an NB-distributed RV, $\mathbf{X}^{m'}$ using Gaussian copula [127], incorporating spatial dependency between the observations via a kernel covariance matrix $H$ with an exponential kernel on the $L^2$ distances and varying lengthscale ($l$) parameters [114]. Then, based on the simulated $\mathbf{X}^{m'}$, we generate

$\mathbf{X}^m$ following Eq. 2 with a fixed slope $\nu$. More specifically, for a fixed choice of $l$, failure probability $\psi_{m'}$, dispersion parameter $r_{m'}$ for variable $m'$, and dispersion parameter $r_m$ for variable $m$, we consider the following steps

1. Simulate a spatially autocorrelated normal RV of size $n$ using a Gaussian process (GP) model:

$$\mathbf{Z}^{m'} \sim MVN\left(\mathbf{0}, H\right), \quad H_{k_1k_2} = \exp\left(-\frac{|s_{k_1} - s_{k_2}|}{l}\right),$$

2. Transform to a vector of uniform RVs using the standard normal CDF ($\Phi$):

$$\mathbf{U}^{m'} = \Phi(\mathbf{Z}^{m'}),$$

3. Convert to a vector of NB RVs using the inverse CDF of $NB(\psi_{m'}, r_{m'})$, denoted by $F^{-1}_{NB(\psi_{m'},r_{m'})}$:

$$\mathbf{X}^{m'} = F^{-1}_{NB(\psi_{m'},r_{m'})}(\mathbf{U}^{m'}),$$

Each element of the resulting vector $\mathbf{X}^{m'}$ retains the marginal NB distribution, $NB(\psi_{m'}, r_{m'})$, where $\psi_{m'}$ is the failure probability and $r_{m'}$ is the dispersion.

4. Generate the link function to simulate variable $m$ with a fixed slope of $\beta_1^{mm'}(s_k) = \nu$ (2):

$$\boldsymbol{\eta}_m = \boldsymbol{\beta}_0^{mm'} + \nu \log(\mathbf{X}^{m'} + 1), \quad \boldsymbol{\beta}_0^{mm'} \sim MVN\left(\mathbf{0}, 0.5H\right),$$

5. Convert the link vector to failure probabilities $\boldsymbol{\psi}_m$ and simulate $\mathbf{X}^m$ from the NB distribution:

$$\boldsymbol{\psi}_m = \frac{\exp(\boldsymbol{\eta}_m)}{1 + \exp(\boldsymbol{\eta}_m)}, \quad \mathbf{X}^m \sim NB(\boldsymbol{\psi}_m, r_m)$$

where $r_m$ is a prefixed dispersion parameter. The $k$-th element of $\mathbf{X}^m$ follows $NB(\psi_{mk}, r_m)$, where $\boldsymbol{\psi}_m = (\psi_{m1}, \ldots, \psi_{mn})^T$ and $\boldsymbol{\eta}_m = (\eta_{m1}, \ldots, \eta_{mn})^T$.

Three values of the lengthscale $l$ are considered, $l = 3.6, 7.2, 18$, with the corresponding structure of $H$ displayed in Fig. 3A. The failure probability of variable $m'$ and dispersion parameters are kept fixed, $\psi_{m'} = 0.5$, $r_m = r_{m'} = 1$. The slope parameter $\nu$ is varied between $\{-0.75, -0.5, -0.25, 0, 0.25, 0.5, 0.75\}$, with negative and positive values representing negative and positive association, respectively. Higher absolute value of $\nu$ dictates the strength of association, and $\nu = 0$ corresponds to the null model, i.e., $\mathbf{X}^m$ and $\mathbf{X}^{m'}$ are independent.

##### 4.4.2 Simulation design 2

In this design, $(\mathbf{X}^m, \mathbf{X}^{m'})$ are simulated jointly using a bivariate Gaussian copula and spatial dependency incorporated using a bivariate GP framework, where the joint covariance matrix has a Kronecker product structure, comprising a $2 \times 2$ correlation matrix and the distance kernel covariance matrix $H$ (Eq. 9.11 from Banerjee et al. (2014)[97] and Eq. 6 from the Supplementary Material). Specifically, we consider the following steps

1. Simulate spatially cross-correlated normal RVs:

$$(\mathbf{Z}^m, \mathbf{Z}^{m'})^T \sim MVN\left(\begin{bmatrix}\mathbf{0}\\ \mathbf{0}\end{bmatrix}, \mathbf{\Sigma} = \begin{bmatrix}1 & \nu \\ \nu & 1\end{bmatrix} \otimes H\right), \quad H_{k_1k_2} = \exp\left(-\frac{|s_{k_1} - s_{k_2}|}{l}\right),$$

2. Transform to uniform RVs using the standard normal CDF ($\Phi$):

$$\mathbf{U}^m = \Phi(\mathbf{Z}^m), \quad \mathbf{U}^{m'} = \Phi(\mathbf{Z}^{m'}),$$

3. Convert to NB random variables using the inverse CDFs:

$$\mathbf{X}^m = F^{-1}_{NB(\psi_m, r_m)}(\mathbf{U}^m), \quad \mathbf{X}^{m'} = F^{-1}_{NB(\psi_{m'}, r_{m'})}(\mathbf{U}^{m'}),$$

Each element of the resulting vectors $\mathbf{X}^m$ and $\mathbf{X}^{m'}$ retain the marginal NB distributions, $NB(\psi_m, r_m)$ and $NB(\psi_{m'}, r_{m'})$, respectively, where $\psi_m, \psi_{m'}$ are failure probabilities and $r_m, r_{m'}$ are dispersions.

The lengthscale $l$ is varied between $\{0.6, 1.8, 3.6, 7.2\}$. The failure probabilities and dispersion parameters are kept fixed, $\psi_m = \psi_{m'} = 0.5$, $r_m = r_{m'} = 1$. The parameter $\nu$ is varied between $\{-0.75, -0.5, -0.25, 0, 0.25, 0.5, 0.75\}$, with similar implications on the direction and strength of association as before.

#### 4.5 Comparison Methods

We compare SpaceBF to five different methods: 1) MERINGUE [98] with the Delaunay triangulation network, 2) SpatialDM [102] with the kernel weight matrix (lengthscale 1.2 as used in the original paper for the melanoma dataset), 3) Lee's $L$ [110] with $k$-NN network (implemented in $R$ package spdep [206], $k = 1$), 4) SpaGene [100], and 5) PearsonCorr, which is the simple Pearson's correlation. We do not use the original packages of LIANA+ [104] and Voyager [117], as the former essentially implements SpatialDM and the latter is a direct application of Lee's $L$. Table 1 summarizes the methods in terms of their assumptions and limitations. We also attempted to evaluate the performance of SpatialCorr [175] and Copulacci [105]. SpatialCorr was straightforward to use, but it proved highly sensitive to the choice of the lengthscale $l$ in its innovative use of the spatial covariance matrix $H$. In contrast, Copulacci was challenging to use, with limited GitHub documentation and no follow-up from the authors.

| Method | Central metric or idea | Global and local association tests | Test type | Potential sensitivity |
|---|---|---|---|---|
| MERINGUE | Bivariate Moran's $I$ | Global | Permutation test | Neighborhood network |
| SpatialDM | Bivariate Moran's $I$ | Global and local | Permutation or exact test | Lengthscale in the kernel weight matrix |
| LIANA+ | Bivariate Moran's $I$ and cosine similarity | Global and local | Permutation test | As above |
| Voyager | Lee's $L$ | Global and local | Permutation test | Neighborhood network |
| SpaGene | k-NN network and earth mover's distance | Global but with local interaction scores | Permutation test | Choice of $k$ |
| PearsonCorr | Pearson's correlation | Global | Permutation or exact test | None |

Table 1: Comparison of the methods in terms of the underlying assumptions.

### 5 Data and code availability

The datasets analyzed are publicly available from the links provided in the corresponding manuscripts: 1) cutaneous melanoma [39] and 2) cSCC [169]. A GitHub $R$ package implementing the proposed method and the datasets in ".RData" format will be made available soon at https://github.com/sealx017/SpaceBF/.

### 6 Acknowledgment

S.S. and B.N. were supported in part by the Biostatistics Shared Resource, Hollings Cancer Center, Medical University of South Carolina (P30 CA138313).
