# SpaceBF: Spatial coexpression analysis using Bayesian Fused approaches in spatial omics datasets

**DOI:** 10.1101/2025.03.29.646124

**Published:** 2025-06-10

**Authors:** Souvik Seal, Brian Neelon

## Abstract

Advancements in spatial omics technologies have enabled the measurement of expression profiles of different molecules, such as genes (using spatial transcriptomics), and peptides, lipids, or N-glycans (using mass spectrometry imaging), across thousands of spatial locations within a tissue. While identifying molecules with spatially variable expression is a well-studied statistical problem, robust methodologies for detecting spatially varying co-expression between molecule pairs remain limited. To address this gap, we introduce a Bayesian fused modeling framework for estimating molecular coexpression at both local (location-specific) and global (tissue-wide) levels, offering a refined understanding of cell-cell communication (CCC) mediated through ligand-receptor and other molecular interactions. Through extensive simulations, we demonstrate that our approach, termed SpaceBF, achieves superior specificity and power compared to existing methods that predominantly rely on geospatial metrics such as bivariate Moran's I and Lee's L. Applying our framework to real spatial transcriptomics and proteomics datasets, we uncover novel biological insights into molecular interactions across different cancer types.

## Full Text Availability

The license terms selected by the author(s) for this preprint version do not permit archiving in PMC. The full text is available from the preprint server.

