# SpaceBF: Spatial coexpression analysis using Bayesian Fused approaches in spatial omics datasets

**DOI:** 10.1101/2025.03.29.646124

**Published:** 2025-06-22

**Authors:** Souvik Seal, Brian Neelon

**Affiliations:** Department of Public Health Sciences, College of Medicine, Medical University of South Carolina, Charleston, USA

**Keywords:** Spatial co-expression, CCC, Bayesian fusion, Horseshoe prior, Bivariate association, Spatial transcriptomics

## Abstract

Advancements in spatial omics technologies have enabled the measurement of expression profiles of different molecules, such as genes (using spatial transcriptomics), and peptides, lipids, or N-glycans (using mass spectrometry imaging), across thousands of spatial locations within a tissue. While identifying individual molecules with spatially variable expression is a well-studied statistical problem, robust methodologies for detecting spatially varying co-expression between molecule pairs remain limited. To address this gap, we introduce a Bayesian fused modeling framework for estimating molecular co-expression at both local (location-specific) and global (tissue-wide) levels, offering a refined understanding of cell-cell communication (CCC) mediated through ligand-receptor and other molecular interactions. Through extensive simulations, we demonstrate that our approach, termed SpaceBF, achieves superior specificity and power compared to existing methods that predominantly rely on geospatial metrics such as bivariate Moran’s I and Lee’s L. Applying our framework to real spatial transcriptomics and proteomics datasets, we uncover novel biological insights into molecular interactions across different cancers.

## 1 Introduction

Technological advances in spatial omics [1, 2, 3] have enabled *in situ* profiling of varying molecules, including genes (via spatial transcriptomics (ST)) [4, 5, 6, 7], lipids or peptides (using mass spectrometry imaging (MSI)) [8, 9, 10, 11], and immune proteins (through multiplex immunofluorescence (mIF)) [12, 13, 14, 15], within tissues. The technologies offer distinct yet complementary biological insights, differing in spatial resolution and the number of detectable molecules (throughput). For example, the next-generation sequencing (NGS)-based ST platform Visium (from 10X Genomics) [16] offers transcriptome-wide gene-expression profiling (throughput ~ 20,000) at a 55 *μm* spot-level resolution. MALDI MSI-based platforms (from Bruker Daltonics [17] and others) offer profiling different types of molecules, such as peptides, lipids, nucleotides, proteins, metabolites, and N-glycans, (throughput ~ 50–1000) at 10 *μm* spot-level resolution. The mIF platform PhenoCycler (from Akoya Biosciences) [18] enables protein profiling (throughput ~ 40) at a 0.6 *μm* cellular resolution. Despite these differences, the underlying data structure remains largely consistent across technologies and platforms, comprising a collection of spatial locations (from single or multiple samples) with observed expression or intensity of various molecules. Consequently, common biostatistical questions arise, centering the spatial dynamics of molecules within the complex tissue or tumor microenvironment (TME) [19, 20, 21, 22, 23].

In the context of ST datasets, identifying spatially variable genes (SVGs), i.e., the genes exhibiting spatially structured expression patterns across the tissue, has gained significant attention [24, 25, 26, 27, 28, 29, 30, 31, 32, 33, 34, 35, 36, 37, 38]. It enables critical downstream analyses such as discovering potential biomarkers and defining tissue regions that influence cellular differentiation and function [39, 40, 41, 42]. Analogously, for mIF or imaging mass cytometry (IMC) datasets, innovative methods [43, 44, 45, 46, 47, 48, 49, 50, 51] have been proposed to understand the spatial distribution of immune cell types (defined by binarizing the expression profile of immune proteins) across the TME. Building upon this univariate framework, which typically analyzes one molecule at a time, another widely investigated problem has been spatial domain detection, i.e., deconvolving the tissue into distinct, spatially contiguous neighborhoods based on multivariate gene expression (ST) [52, 53, 54, 55, 56, 57, 58, 59, 60, 61, 62, 63] or immune cell type composition (mIF) [64, 65, 66, 67, 68, 69]. It aids mapping the molecular and functional landscape of tissues, elucidating disease progression, and guiding targeted therapies [70, 71, 72]. While some of the referenced methods can be adapted for use with MSI datasets, it is important to underscore the lack of sophisticated spatial functionalities of the existing bioinformatics toolboxes [73, 74, 75, 76].

While univariate and multivariate spatial analyses have garnered significant attention, a critical intermediate task remains underexplored: bivariate spatial co-expression analysis of molecular pairs at both “local” (spot/cell-specific) and “global” (tissue-wide) levels, aimed at precisely characterizing the spatial interaction or binding pattern of any two molecules throughout the tissue plane. To emphasize the importance of such an analysis, we review the concepts of cell-cell communication (CCC) [77, 78, 79, 80]. CCC is a fundamental biological process through which cells exchange information via direct contact or signaling molecules (ligands) binding to receptor molecules present on the same or different cells. It regulates essential biological functions, including tissue development [81] and immune responses [82], and its disruption has been implicated in the onset and progression of cancer [83]. Autocrine, juxtacrine, and paracrine signaling are three major pathways of CCC [84]. In autocrine signaling, ligands released by a cell bind to receptors on the same cell, while in juxtacrine and paracrine signaling, the ligands target adjacent and nearby cells. The study of ligand-receptor interactions (LRI), which involves identifying gene pairs (ligands and receptors) that show coordinated upregulation or downregulation across groups of cells, has become a fundamental approach for inferring CCC from single-cell RNA sequencing (scRNA-seq) datasets [85, 86, 87, 88, 89, 90, 91, 92, 93]. However, these approaches are prone to false positive interactions due to the lack of spatial context in scRNA-seq datasets, treating distant cell pairs similarly to nearby ones [94, 95, 96], which potentially leads to an overestimation of juxtacrine and paracrine signaling. ST datasets offer a natural avenue for improvement by enabling spatially constrained LRI analysis.

A limited number of tools exist for spatial LRI analysis or, more broadly, for assessing bivariate spatial co-expression of molecules in ST or MSI datasets. It should be emphasized that bivariate co-expression can manifest in two ways: (a) joint over- or under-expression within the same cells (correlation) and (b) joint over- or under-expression in neighboring cells (cross-correlation [97]). Some relevant methods include MERINGUE [98], Giotto [99], SpaGene [100], SpaTalk [101], SpatialDM [102], CellChat V2 [103], LIANA+ [104], and Copulacci [105]. We skip the approaches that jointly analyze multiple LR pairs [106, 107]. Methods such as MERINGUE, Giotto, and SpaTalk provide only a global summary of spatial co-expression across a tissue, whereas others also offer local (spot/cell-specific) estimates. Let the standardized expression of two genes $\left(m,m^{\prime }\right)$ be $X^{m}(s)$ and $X^{m^{\prime }}(s)$ at location s for $s\in \left\{s_{1},\ldots ,s_{n}\right\}$, and $X^{m}={\left(X^{m}\left(s_{1}\right),\ldots ,X^{m}\left(s_{n}\right)\right)}^{T}$, $X^{m^{\prime }}={\left(X^{m^{\prime }}\left(s_{1}\right),\ldots ,X^{m^{\prime }}\left(s_{n}\right)\right)}^{T}$. For a global summary of spatial co-expression, MERINGUE and SpatialDM leverage a popular geospatial metric termed the bivariate Moran’s $I\;(I_{BV}$) [108, 109], interpreted as the Pearson correlation between one variable and the spatial lagged version of the other [110, 111, 112]. Mathematically, $I_{BV}\propto {\left(X^{m}\right)}^{T}WX^{m^{\prime }}$, where $W=\left[\left[w_{k_{1}k_{2}}\right]\right]$ is the spatial weight matrix that controls the spatial lagging. As W, MERINGUE uses a binary adjacency matrix based on the Delaunay triangulation [113] of the spatial locations ($w_{k_{1}k_{2}}=1$ if locations ($s_{k_{1}},s_{k_{2}}$) are connected, or 0 otherwise). SpatialDM uses a kernel covariance matrix or Gram matrix [114] based on the $L_{2}$ distance between locations ($w_{k_{1}k_{2}}=k_{l}({\left|s_{k_{1}}-s_{k_{2}}\right|}^{2})$, where $k_{l}$ is a kernel function with lengthscale parameter l [115]). For local estimates of spatial correlation, SpatialDM considers the bivariate local Moran’s $I\left(I_{BV}^{local}(s)\right)$ based on the local indicators of spatial association (LISA) approach [116]. The LIANA+ toolbox implements SpatialDM and introduces a similar spatially weighted cosine similarity index. Of note, a newer package named Voyager [117] considers Lee’s L statistic [110], which has a slightly different formulation than $I_{BV}$. A critical yet often overlooked aspect of ST data analysis is that gene expression, measured in terms of unique molecular identifier (UMI) count, is inherently a discrete random variable (RV). However, the above methods assume normality upon a variance-stabilizing transformation [118, 119, 120], which may obscure true signals and have been widely criticized both within the ST literature [26, 55, 121, 122] and in broader contexts [123, 124, 125, 126]. Addressing this issue, Copulacci models a pair of genes as bivariate Poisson-distributed RVs, with their correlation in spatially adjacent cells captured using a Gaussian copula [127]. For inference, these methods typically rely on a permutation test [128].

Bivariate Moran’s $I\;\left(I_{BV}\right)$ and Lee’s L statistic, as implemented in MERINGUE, SpatialDM, LIANA+, and Voyager, are primarily recommended as exploratory metrics for assessing cross-correlation rather than as rigorous hypothesis testing tools [129, 97], in traditional spatial statistical literature. In simulation studies (see Section 2.2), we have shown that even when two variables are independently simulated with certain spatial covariance structures, the unmodeled spatial autocorrelation introduces a confounding effect on the bivariate association, leading to significantly inflated type 1 error rates. A similar issue is well documented, as extensive literature highlights the limitations of using simple Pearson correlation to assess dependencies between two variables in the presence of spatial autocorrelation [130, 131, 132, 133, 134]. By extension, since $I_{BV}$ and Lee’s L are both fundamentally based on Pearson correlation between spatially lagged variables, they may be susceptible to similar pitfalls. Furthermore, these spatially weighted association indices, being model-free, are unable to seamlessly adjust for cell-level covariates such as cell type, a limitation also present in Copulacci. As a side note, mapping to the aforementioned CCC pathways, Pearson correlation between ligand and receptor can be interpreted as a proxy for autocrine signaling, while cross-correlation may reflect a combination of juxtacrine and paracrine signaling.

We approach the bivariate spatial co-expression detection as a generalized linear regression problem, modeling a molecule m as the outcome and the other molecule $m^{\prime }$ as the predictor (see Section 4). For ST datasets, gene expression or UMI count is modeled as an overdispersed negative binomial (NB)-distributed RV [135], while an alternative Gaussian model is considered for continuous cases. The regression coefficients, both intercept $\left(\beta_{0}^{mm^{\prime }}(s)\right)$ and slope $\left(\beta_{1}^{mm^{\prime }}(s)\right)$, are assumed to vary across locations (s) exhibiting spatial dependency. Known as the spatially varying coefficients (SVC) model [136], this framework provides exceptional flexibility and precision in capturing locally changing co-expression patterns through $\beta_{1}^{mm^{\prime }}\left(s\right)$. A large positive $\beta_{1}^{mm^{\prime }}(s)$ suggests strong positive co-expression at location s, i.e., joint up or down-regulation, whereas a large negative value indicates avoidance or repulsion. The average of $\beta_{1}^{mm^{\prime }}(s)$’s, $\bar{\beta_{1}^{mm^{\prime }}}=\sum_{k}\;\beta_{1}^{mm^{\prime }}\left(s_{k}\right)/n$, provides a summary of the global co-expression pattern. Similar models have been widely used in fields such as disease mapping [137, 138], econometrics [139, 140], ecological studies [141, 142], and neuroimaging research [143, 144]. In the Bayesian paradigm, the spatial dependency between $\beta_{0}^{mm^{\prime }}(s)$’s and $\beta_{1}^{mm^{\prime }}(s)$’s is typically modeled using a conditionally autoregressive (CAR) [145, 146, 147] or Gaussian process (GP) priors [148, 97, 149]. In contrast, we introduce a locally adaptive spatial Gaussian Markov random field (GMRF) prior [150] based on the concepts of fusion penalties [151, 152, 153] and horseshoe prior [154, 155, 156], extending a related work in the frequentist setup [157]. Briefly, the prior incorporates the spatial similarity between two adjacent locations, $\left(s_{k_{1}},s_{k_{2}}\right)$, by encouraging $|\beta_{0}^{mm^{\prime }}\left(s_{k_{1}}\right)-\beta_{0}^{mm^{\prime }}\left(s_{k_{2}}\right)|\approx 0$ and $|\beta_{1}^{mm^{\prime }}\left(s_{k_{1}}\right)-\beta_{1}^{mm^{\prime }}\left(s_{k_{2}}\right)|\approx 0$. Adjacency is defined using the minimum spanning tree (MST) [158, 159, 160] constructed with the $L_{2}$ distances between locations as edge weights. The MST of a connected, weighted graph offers the most economical connectivity between all vertices without cycles, minimizing the total edge weight. A detailed discussion on the choice of MST as the underlying spatial graph, along with its implications for the resulting GMRF prior, is provided. The proposed method, SpaceBF, is evaluated against existing approaches using realistic simulation scenarios, showcasing its high specificity and power. It is further applied to three real datasets: a) an ST dataset on cutaneous melanoma [39] for spatial LRI analysis, b) an ST dataset on cutaneous squamous cell carcinoma [161] for keratin-interaction analysis, and c) a spatial proteomics dataset on ductal carcinoma in situ (DCIS) from the Medical University of South Carolina (MUSC) for peptide co-localization analysis.

## 2 Result

### 2.1 Real data analysis

#### 2.1.1 Melanoma ST dataset

**Figure 1:**
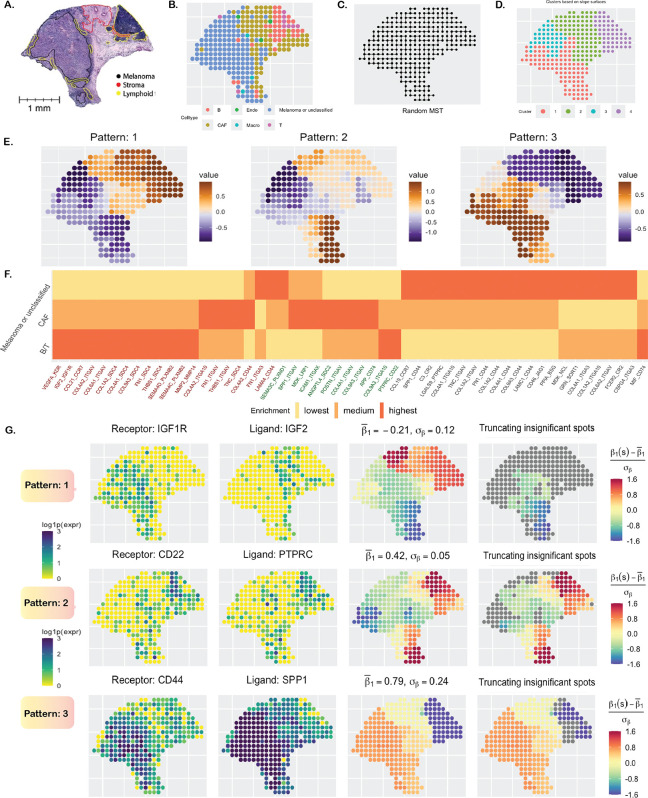
Cutaneous melanoma data analysis. **A**. Annotated H&E-stained image. **B**. Cell types based on gene expression. **C**. Minimum spanning tree (MST) capturing the spatial structure. **D**. Clustering of spots based on centered and scaled estimates of slope surfaces of 53 statistically significant LR pairs. **E**. The three main spatial patterns of the estimated surfaces. **F**. Enrichment of LR interactions in three major cell types, with LR names arranged and color-coded according to their respective patterns. **G**. The first two columns show the expression of three LR pairs. The third column displays the centered and scaled slope surfaces. In the fourth column, insignificant spot-level slope estimates are greyed.

We analyzed a cutaneous melanoma dataset [39] from a long-term survivor (10+ years), collected using the ST technology [4], comprising 293 spots, each 100 *μm* in size and at a 200 *μm* center-to-center distance. There are 16,148 genes, forming 1,180 known ligand-receptor (LR) pairs as available from CellChatDB [88]. There are three major pathologist-annotated regions as seen in the histology image (Fig. 1A), collected from Thrane et al. (2018), and 6 major cell types (Fig. 1B) predicted using the RCTD [162] package based on overall gene expression [102]. After filtering out genes with extremely low expression (< 0.2 × 293 ≈ 59 reads), 161 LR pairs remain, which were examined using our method SpaceBF. To briefly summarize the SpaceBF workflow, it first constructs an MST based on the spatial coordinates of the spots (Fig. 1C). Then, for every LR pair: ($m^{\prime },m$), it considers Eq. 2 with the receptor expression as $X^{m}\left(s_{k}\right)$ and the ligand expression as $X^{m^{\prime }}\left(s_{k}\right)$, and $s_{k}$ representing a spot. Following parameter estimation via a Markov Chain Monte Carlo (MCMC) procedure, the framework performs two hypothesis tests to assess the significance of spatial co-expression at both global and local levels (see Section 4.3). Using the global test in this dataset, SpaceBF identified 53 LR pairs at a significance level 0.05 (33 at an FDR of 0.1). The estimated slope surface $\beta_{1}^{mm^{\prime }}\left(s_{k}\right)$ of different LR pairs exhibits distinct patterns. To highlight these differences, we classify the detected LR pairs into 3 major patterns (Fig. 1E) based on hierarchical clustering [163] of the standardized vector $\beta_{1}^{mm^{\prime }*}={\left(\beta_{1}^{mm^{\prime }}\left(s_{1}\right)-\bar{\beta_{1}^{mm^{\prime }}},\ldots ,\beta_{1}^{mm^{\prime }}\left(s_{n}\right)-\bar{\beta_{1}^{mm^{\prime }}}\right)}^{T}/\sigma_{\beta }^{mm^{\prime }}$, where $\bar{\beta_{1}^{mm^{\prime }}}=\sum_{k}\;\beta_{1}^{mm^{\prime }}\left(s_{k}\right)/n$ and $\sigma_{\beta }^{mm^{\prime }}$ are the tissue-wide average and the SD of estimated $\beta_{1}^{mm^{\prime }}\left(s_{k}\right)$’s, respectively. 20 LR pairs follow pattern 1, while 22 and 11 LR pairs correspond to patterns 2 and 3, respectively. Similarly, the spots are grouped into 4 clusters based on the spot-level vectors of slopes corresponding to the 53 detected LR pairs (Fig. 1D). It is evident that clusters 1 and 3 correspond to the melanoma region, while clusters 2 and 4 loosely correspond to the stroma and lymphoid regions, respectively. Returning to the LR patterns, in Fig. 1F, the LR pairs are arranged sequentially from pattern 1 to 3, highlighting the enrichment of their interaction in three major cell types. For example, $\sum_{k\in B/T\;\text{cells}\;}\;\beta_{1}^{mm^{\prime }*}\left(s_{k}\right)$ represents the enrichment within B/T cells relative to the average enrichment $\bar{\beta_{1}^{mm^{\prime }}}$ and scaled by the SD. The levels “highest,” “medium,” and “lowest” indicate the degree of enrichment, with “highest” corresponding to the greatest or most positive enrichment and so on. The majority of LR pairs following pattern 1 exhibit higher or more positive interaction in B/T cells within the lymphoid region (some in CAF cells) and more negative interaction (avoidance or repulsion) in the melanoma region or cells. Pattern 2 mostly corresponds to LR pairs with the highest enrichment in CAF cells, while pattern 3 clearly corresponds to the pairs with the highest enrichment in melanoma cells. Next, we investigate the biological relevance of the estimated slope surfaces for a selected set of LR pairs. The LR pair (IGF2, IGF1R) [164] corresponds to pattern 1 and demonstrates a negative association overall, with an estimated average slope of $\bar{\beta_{1}^{mm^{\prime }}}=-0.212$, and the *p*-value = 0.024, which is consistent with a visual inspection (Fig. 1G). It could indicate a lack of binding between these genes, which would be a generally favorable factor for the survivor [165]. Setting the insignificant $\beta_{1}^{mm^{\prime }*}\left(s_{k}\right)$ values to 0 based on the local test, the negative interaction found in the melanoma region has the highest credibility. The pair (PTPRC, CD22) [166] follows pattern 2, with $\bar{\beta_{1}^{mm^{\prime }}}=0.422$ and *p*-value of 6.28 × 10^−6^. PTPRC, also known as CD45, is a facilitator of T-cell receptor (TCR) and B-cell receptor (BCR) signaling [167], while CD22 is primarily an inhibitor of BCR signaling [168]. Their overall positive co-expression, particularly in the lymphoid region, is likely associated with a balanced B cell regulation, helping to prevent autoimmunity and promoting lymphoid growth in other regions as part of the immune response. The final LR pair we discuss is (SPP1, CD44) [169], which follows pattern 3, exhibiting a highly positive overall co-expression with $\bar{\beta_{1}^{mm^{\prime }}}=0.79$ and *p*-value of 1.03 × 10^−6^. This strong interaction displays a decreasing gradient from the melanoma region to the lymphoid region, which aligns with its known role in dysregulated cytoskeletal remodeling [170], facilitating melanoma cell invasion into surrounding tissues.

#### 2.1.2 cSCC ST dataset

**Figure 2:**
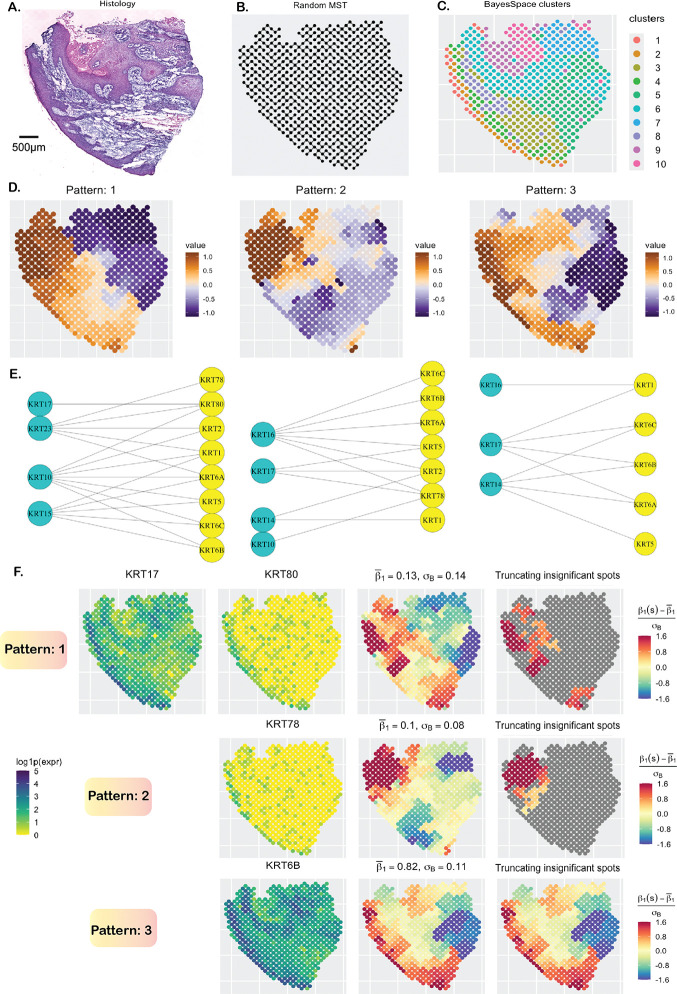
Cutaneous squamous cell carcinoma data analysis. **A**. H&E-stained image. **B**. MST capturing the spatial structure. **C**. Spatial clusters obtained using the BayesSpace package. **D**. The three main spatial patterns of the estimated surfaces. **E**. Bipartite graphs between type 1 and type 2 keratins based on their spatial pattern. **F**. Study of the binding between the type 1 keratin KRT17 and three different type 2 keratins, with each slope surface exhibiting a unique spatial pattern. The insignificant spot-level slope estimates are greyed in the last column.

We analyzed a cutaneous squamous cell carcinoma (cSCC) dataset [161] on a patient sample with a histopathologic subtype of “moderately differentiated” cSCC [171]. The dataset was collected using the ST technology with 621 spots, each of size 110 *μm* and a center-to-center distance of 150 *μm*. There are 16, 643 genes of which 45 are keratins (14 after filtering low-count genes, < 0.2 × 621 ≈ 124 reads). These keratins can be classified into two types: 1) type 1, which includes KRT10, KRT14–KRT17, and KRT23, and 2) type 2, which includes KRT1, KRT2, KRT5, KRT6A, KRT6B, KRT6C, KRT78, and KRT80. The keratins pair together to form intermediate filaments, providing structural support to epithelial cells [172]. In the context of cSCC and other carcinomas, keratins are emerging as highly significant targets for therapeutic intervention [173, 174, 175]. Of note, some of the keratins belong to the GO term: “keratinocyte differentiation” (GO:0030216) and were reported to exhibit strong spatial correlation in an earlier work [176] involving the same dataset. We utilized SpaceBF to investigate the binding between type 1 and type 2 keratins, resulting in a set of 48 keratin pairs. In the histology image (Fig. 2A), the deep blue areas at the top and left sides correspond to tumor regions, while the whitish region at the bottom represents a non-tumor region possibly composed of keratinized layers and stroma [177]. However, the tumor and non-tumor regions are not clearly delineated, a feature characteristic of moderately differentiated cSCC, though the spatial clusters obtained using the BayesSpace package [52] on the transcriptome-wide gene expression profile (Fig. 2C) partially elucidate this distinction. The constructed MST is shown in Fig. 2B. Using the global test, SpaceBF identified 39 keratin pairs at a significance level of 0.05 (41 at an FDR < 0.1), suggesting that most pairs bind to each other, albeit to varying degrees. Similar to the earlier analysis, we classify the detected slope surfaces into 3 major patterns (Fig. 2D) based on hierarchical clustering of the standardized vector $\beta_{1}^{mm^{\prime }*}$. We represent the keratin pairs as bipartite graphs between type 1 and 2 keratins under each pattern (Fig. 2E). One important observation is that type 2 keratins KRT6A, KRT6B, and KRT6C are isoforms of keratin 6 [178] and thus, co-express highly, meaning their binding patterns with any specific type 1 keratin should be similar, as correctly identified by SpaceBF. For instance, the slope surfaces of KRT10 with KRT6A, 6B, and 6C all align with pattern 1, while the slope surfaces of KRT16 with KRT6A, 6B, and 6C all correspond to pattern 2. This consistency underscores the reliability of SpaceBF in identifying true local patterns. In Fig. 2F, we present the estimated slopes for KRT17, which is a well-established therapeutic target in various cancers [179, 180, 181], binding with three type 2 keratins: KRT80 (pattern 1, *p*-value = 0.006), KRT78 (pattern 2, *p*-value = 0.03), and KRT6B (pattern 3, *p*-value = 5.59 × 10^−6^). Notably, the average slope estimates for KRT17-KRT80 and KRT17-KRT78 interactions are small (≈ 0.1), whereas for KRT17-KRT6B, the average slope is substantially higher at 0.82, with the highest local estimates observed mostly in tumor regions. These trends are also evident from the individual expression profiles provided in Fig. 2F. Although the expression patterns of KRT80 and KRT78 appear similar, a closer examination reveals that KRT80 exhibits a thicker band of expression on the left, specifically within the tumor regions. This distinction contributes to the difference in co-expression patterns of KRT17-KRT80 and KRT17-KRT78. As previously noted, both association levels are low, also indicated by the small number of significant spots identified by the local test, 72 and 49, respectively.

#### 2.1.3 DCIS proteomics dataset

**Figure 3:**
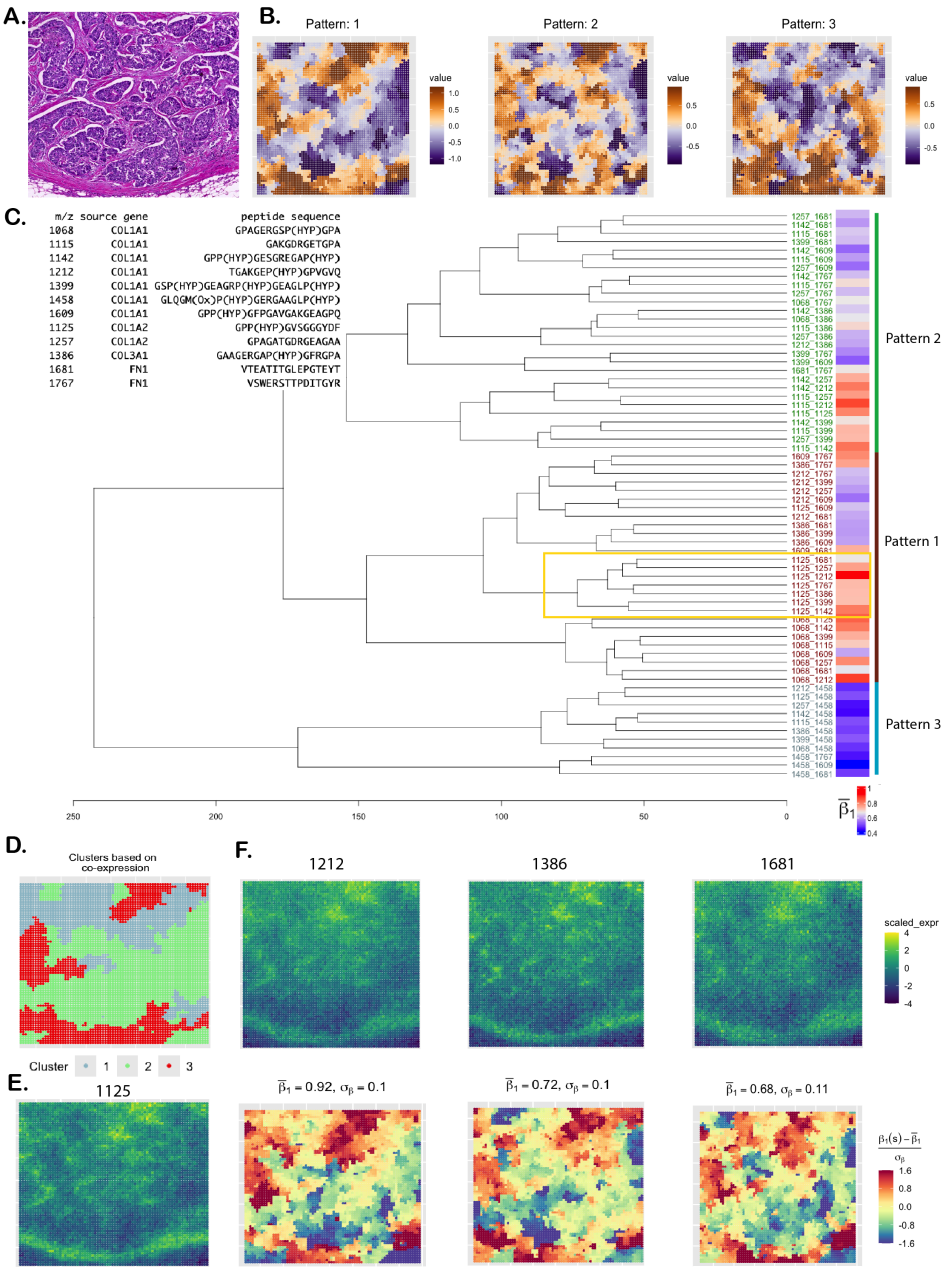
DCIS data analysis. **A**. H&E-stained image. **B**. Patterns of standardized co-expression (slope) of 66 peptide pairs (from 12 peptides). **C**. Peptide description and dendrogram corresponding to the patterns. Mean slope estimates are presented as a heatmap on the right. **D**. Clustering of spots based on the slope surfaces. **E**. Scaled expression of the peptide 1125 forming the yellow-bordered module in the dendrogram. **F**. Scaled expression of three peptides belonging to the same module (top row) and their spatial co-expression with peptide 1125 (bottom row).

We analyzed a single-sample ductal carcinoma in situ (DCIS) dataset collected using the MALDI MSI spatial proteomics platform, as part of an ongoing study at the MUSC aimed at defining the proteomic landscape of DCIS and invasive breast cancer (IBC), in terms of collagen peptides and immune cell types. DCIS is marked by the abnormal growth of malignant epithelial cells confined to the breast’s milk ducts, without invading the surrounding stromal tissue. While prognosis is excellent, around 20–40% of diagnosed DCIS progress to IBC [182, 183]. Understanding proteomic co-localization within the extracellular matrix (ECM) of a DCIS tissue is crucial for assessing progression risk and predicting therapeutic response, as the ECM plays a key role in regulating tumor cell proliferation, migration, and survival [184]. In this dataset, there are 5,548 tissue spots and 12 ECM peptides, whose 122 pairwise interactions were of our interest. As the peptide expression is continuous-valued, we used the Gaussian model of SpaceBF for this analysis (Eq. 1). Seven of the peptides are derived from the COL1A1 gene, while the remaining peptides originate from COL1A2, COL3A1, and FN1 (Fig. 3C). From the histology image (Fig. 3A), the stromal ECM can be identified by the light pink staining of fibrous connective tissue, while epithelium regions are highlighted in deep blue. Using hierarchical clustering, we group the standardized slope surfaces $\left(\beta_{1}^{mm^{\prime }*}\right)$ into three patterns (Fig. 3B), and the spots into three clusters based on the spot-level vectors of standardized slopes (Fig. 3D). While the differences among the three patterns are subtle, the spot clusters are well-defined and spatially distinct: the red cluster (cluster 3) aligns with stromal regions, the green cluster (cluster 2) with epithelial regions, and the light blue cluster (cluster 1) is a mixture of both. It is important to note that the MSI image has substantially lower resolution compared to the histology image, making one-to-one correspondence between the two inherently challenging. Patterns 1 and 2 (Fig. 3B), which visually resemble each other, both suggest strong co-localization of the associated peptide pairs in the stroma. This is expected, as all of these peptides are known to constitute the stromal ECM. The tree diagram in Fig. 3C shows the hierarchical relationships between the peptide pairs, with their patterns indicated on the right. The module highlighted by the yellow box includes pairs involving peptide 1125 (from COL1A2) and 7 other peptides. From Figs. 3E and 3F (top row), peptides 1125, 1212, 1386, and 1681 (from the module) show pronounced co-expression in the stromal region. Correspondingly, the estimated slope surfaces (Fig. 3F, bottom row) for the pairs (1125, 1212), (1125, 1386), and (1125, 1681) all fall under pattern 1, but the association strength is notably higher for (1125, 1212): $\bar{\beta_{1}^{mm^{\prime }}}=0.92$, compared to 0.72 and 0.68 for the other two. Although the existing literature on these interactions is limited, the findings will inform future comparative analyses of ECM compositions across DCIS subtypes and stages of progression [185, 186].

### 2.2 Simulation studies

**Figure 4:**
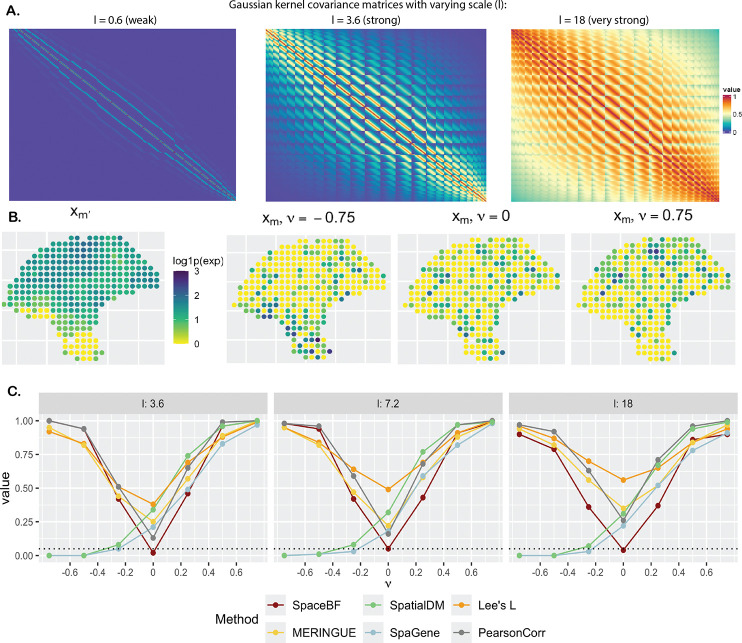
Comparison of global tests under the simulation design 1. **A**. Heatmap of the spatial covariance matrix H with an exponential kernel and $L_{2}$ distance, for varying values of the lengthscale parameter l. **B**. Simulated $X^{m}$ based on Eq. 2, for a fixed $X^{m^{\prime }}$ but different values of the constant slope $\beta_{1}^{mm^{\prime }}(s)=\nu$. **C**. Performance of the methods in terms of type 1 error (ν=0) and power (ν≠0). The dotted line represents the significance level 0.05.

We consider the spatial coordinates (*n* = 293) from the cutaneous melanoma dataset. In simulation design 1, one NB-distributed random variable (RV), $X^{m^{\prime }}$ is generated using a Gaussian copula with a spatial covariance matrix H based on an exponential kernel (for varying lengthscale l) and the $L_{2}$ distance. Another NB-distributed RV, $X^{m}$ is then generated using the NB model from Eq. 2 with a constant slope $\beta_{1}^{mm^{\prime }}(s)=\nu$ and $\beta_{0}^{mm^{\prime }}$ simulated using a Gaussian process (GP) model [97] with the spatial covariance matrix H. More details on the design are provided in Section 4.4.1. From Fig. 4A, we notice how the structure of H changes as the lengthscale l varies. The off-diagonal elements of $H\;\left(H_{k_{1}k_{2}}\right)$ can range between 0 and 1. When l=0.6, only the nearest locations ($k_{1},k_{2}$) exhibit high $H_{k_{1}k_{2}}$ with most of the other values being close to 0. In contrast, for l=18, the majority of location pairs have high $H_{k_{1}k_{2}}(\approx 1)$, inducing an exceptionally strong spatial autocorrelation in both variables. To visibly understand how ν might affect the relationship between $X^{m}$ and $X^{m^{\prime }}$, in Fig. 4B, we show the spatial expression of $X^{m}$ for the same $X^{m^{\prime }}$ but three different values of ν,{−0.75, 0, 0.75}. It is somewhat evident that nonzero ν’s result in a visibly positive or negative association, while ν=0 produces a random pattern of $X^{m}$. In Fig. 4C, we show the type 1 error (ν=0) and power (ν≠0) comparison of the different methods, including SpaceBF, for three values of the lengthscale l. When l=3.6, both variables exhibit considerable spatial autocorrelation, yet SpaceBF maintains the correct type 1 error. In contrast, all other methods suffer from inflated type 1 errors. Notably, simple Pearson correlation, while still inflated, performs better in controlling type 1 error compared to methods based on bivariate Moran’s I or Lee’s L. Although SpaGene does not rely on these traditional metrics, it still fails to control type 1 error. The issue becomes more pronounced as l increases. Notably, Lee’s L exhibits the highest inflation in the majority of cases. SpaceBF also retains a high detection power throughout all three cases. Although the power declines slightly for the largest l, as expected, due to a decrease in effective sample size from increased spatial autocorrelation. In summary, the simulation effectively demonstrates the specificity and power of our method.

In simulation design 2, we generate ($X^{m},X^{m^{\prime }}$) jointly as bivariate spatially correlated NB-distributed RVs. This setup is more complex than the previous one, as the association is non-linear and driven by the Kronecker product-based spatial covariance structure (see Section 4.4.2). The methods, except SpaceBF, perform poorly in terms of the type 1 error for l≥1.8. When the spatial autocorrelation is the weakest (l=0.6), Pearson correlation performs well as expected, while spatially weighted indices still show a slight inflation. SpaceBF achieves controlled type 1 error and steady detection power across varying l’s. As earlier, the power decreases as the effective sample size decreases. Together, these two simulation designs demonstrate SpaceBF’s robustness under complex data generation processes. Finally, we argue that incorporating $\beta_{0}^{mm^{\prime }}(s)$ in SpaceBF accounts for the spatial autocorrelation of variable m, thereby mitigating bias in the association analysis of ($m,m^{\prime }$). As previously noted, Pearson correlation is already recognized to be suboptimal in such scenarios, and spatially weighted indices, essentially Pearson correlation between spatially lagged variables, thus also remain susceptible to spurious detections. Recall that MERINGUE uses a binary spatial weight matrix W based on the Delaunay triangulation, while SpatialDM uses a continuous spatial weight matrix having a similar form as H for a particular choice of the lengthscale. Lee’s L is based on a binary k–NN network in our study. Hence, the performance of these methods could be sensitive to choices of the spatial weight matrices, i.e., different networks or lengthscale l values. SpatialDM and SpaGene focus solely on the joint over-expression of molecules, neglecting joint under-expression. As a result, for most values of ν<0, these methods show almost no detection power.

## 3 Discussion

We have developed a rigorous framework for studying spatial co-expression of a pair of molecules in the context of spatial transcriptomics (ST) and mass spectrometry imaging (MSI) datasets, at both global (tissue-wide) and local (cell/spot-specific) levels. Existing tools mostly rely on two exploratory geospatial metrics, namely, bivariate Moran’s I [108] and Lee’s L [110], which lead to highly spurious association inference as demonstrated by our simulation studies. Our proposed approach, SpaceBF, builds on the widely used spatially varying coefficients model [136], effectively capturing spatial autocorrelation and locally varying co-expression patterns. We introduce a novel spatial GMRF prior based on the minimum spanning tree (MST) network [187, 160] between locations and a fused horseshoe prior [153, 156], extending a recent frequentist framework from a different domain [157]. We elucidate the prior’s theoretical properties and explore its connections to the alternative approaches. Integrating this prior both within a Gaussian linear regression model and a more complex negative binomial regression framework [135], SpaceBF is broadly applicable across various analytical contexts and data types.

We conduct a comprehensive evaluation of the proposed method under challenging simulation scenarios, demonstrating its ability to maintain well-controlled type I error rates alongside strong detection power. In three real-world applications, two spatial transcriptomics (ST) datasets and one mass spectrometry imaging (MSI) dataset, the method exhibits robust performance in identifying biologically meaningful molecular interactions, including ligand-receptor (LR) signaling, keratin binding, and peptide co-localization. Notably, the analysis of the cutaneous melanoma sample reveals spatially variable patterns of cell-cell communication, as assessed through LR interactions. The LR pairs exhibit coordinated over- or under-expression within spatially distinct tissue regions (identified from histology) or specific cell types (inferred from transcriptome-wide gene expression). This level of granular understanding may provide critical insights for developing novel, targeted tissue-specific therapies in broader clinical settings [188, 189, 190].

Utilizing the MST as the underlying spatial graph provides several advantages: 1) its uniqueness, eliminating the need to tune additional hyperparameters such as the lengthscale parameter in Gaussian process models [97], and 2) reduced computational complexity by inducing a sparser precision matrix. In all of our simulation studies, where spatial dependency is consistently generated using a Gaussian process with an exponential kernel and varying lengthscales, the MST-based approach performs exceptionally well, underscoring its robustness. Nevertheless, fixing the spatial structure to a spanning tree may not always be optimal, as it could exclude important edges [191]. Future work will consider treating the spanning tree as an unknown parameter to be iteratively updated [192]. While covariate adjustment is readily accommodated in our framework, further exploration is warranted to better understand cell-type-specific co-expression patterns across diverse datasets. In addition, extending the models to handle multiple spatially varying predictors presents a promising avenue, which we aim to pursue with particular attention to computational scalability and multicollinearity. Although SpaceBF has only been used in the ST and MSI datasets, it could also be useful in multiplex immunofluorescence (mIF) or imaging mass cytometry datasets where the molecular outcome of interest is generally immune cell types. To study cell type co-localization in such cases, one could split an mIF image into regular grids and count how many cells of two types ($m,m^{\prime }$) fall into each grid. Assuming that the grid centers are the locations $s_{k}$’s, an MST can be constructed between them, and the spatial cell counts $X^{m}\left(s_{k}\right)$ and $X^{m^{\prime }}\left(s_{k}\right)$ can be analyzed as before.

## 4 Methods

### 4.1 Gaussian and negative binomial models

We assume a single sample or image with n spots/cells. Let $X^{m}\left(s_{k}\right)$ and $X^{m^{\prime }}\left(s_{k}\right)$ denote the expression of a pair of molecules m and $m^{\prime }$, and $C\left(s_{k}\right)$ be a vector of p covariates observed at spot/cell location $s_{k}$, for k∈{1,…,n}. For example, $C\left(s_{k}\right)$ can be the cell type indicator or a vector of cell type proportions [162]. We consider the following Gaussian spatially varying coefficients (SVC) model [136]

(1) $$X^{m}\left(s_{k}\right)=\beta_{0}^{mm^{\prime }}\left(s_{k}\right)+X^{m^{\prime }}\left(s_{k}\right)\beta_{1}^{mm^{\prime }}\left(s_{k}\right)+C{\left(s_{k}\right)}^{T}\alpha_{m}+\epsilon \left(s_{k}\right),\;k=1,\ldots ,n,$$

where $\beta_{0}^{mm^{\prime }}\left(s_{k}\right)$ and $\beta_{1}^{mm^{\prime }}\left(s_{k}\right)$ denote spatially varying intercept and slope, respectively, $\alpha_{m}$ is a fixed effect vector, and $\epsilon \left(s_{k}\right)$ is an independent error term. To interpret the model, a significantly positive $\beta_{1}^{mm^{\prime }}\left(s_{k}\right)$ implies that the molecules $\left(m,m^{\prime }\right)$ co-express at the location $s_{k}$, while a significantly negativevalue suggests avoidance. Intuitively, $\beta_{0}^{mm^{\prime }}\left(s_{k}\right)$ accounts for spatial autocorrelation of molecule m. More discussion on the underlying bivariate spatial process is provided in the Supplementary Material. For a count-valued $X^{m}\left(s_{k}\right)$ (e.g., genes in the ST datasets), we consider a spatially varying negative binomial (NB) distribution [135] as $NB\left(\psi_{m}\left(s_{k}\right),r_{m}\right)$ with the failure probability $\psi_{m}\left(s_{k}\right)$ modeled following Pillow and Scott (2012) [193],

(2) $$\begin{array}{l} \eta_{m}\left(s_{k}\right)=\beta_{0}^{mm^{\prime }}\left(s_{k}\right)+X^{m^{\prime }}\left(s_{k}\right)\beta_{1}^{mm^{\prime }}\left(s_{k}\right)+C{\left(s_{k}\right)}^{T}\alpha_{m}\\ p\left(X^{m}\left(s_{k}\right)|\psi_{m}\left(s_{k}\right),r_{m}\right)\propto {\left(1-\psi_{m}\left(s_{k}\right)\right)}^{r_{m}}\psi_{m}{\left(s_{k}\right)}^{X^{m}\left(s_{k}\right)},\;\psi_{m}\left(s_{k}\right)=\frac{exp\left(\eta_{m}\left(s_{k}\right)\right)}{1\;+\;exp\left(\eta_{m}\left(s_{k}\right)\right)}, \end{array}$$

where p(.|.) denotes the conditional probability mass function (PMF) and the dispersion parameter $r_{m}(>0)$ is assumed to be constant across locations. To explain how this framework effectively models overdispersion: as $r_{m}\to \infty$, it reduces to a Poisson model; in contrast, as $r_{m}\to 0$, the counts become increasingly dispersed relative to the Poisson distribution 135. Admittedly, this model is limited as the count-valued nature of the molecule $X^{m^{\prime }}\left(s_{k}\right)$ is not prioritized, appearing as a spatially varying predictor. Jointly modeling $\left(X^{m}\left(s_{k}\right),X^{m^{\prime }}\left(s_{k}\right)\right)$ as bivariate NB (BNB) random variables is a possible approach that we do not pursue, as the existing definitions of the BNB distribution (outside of Copula-based constructions) [194, 195, 196, 197] vary considerably, often leading to restrictive correlation structures and inefficient MCMC sampling.

The models in Eqs. 1 and 2 are over-parametrized and do not incorporate spatial dependency between $\beta_{0}^{mm^{\prime }}\left(s_{k}\right)$’s and $\beta_{1}^{mm^{\prime }}\left(s_{k}\right)$’s, which we discuss next. Let G=(V,E) denote the MST network between the locations constructed using the $L_{2}$ distance: ${\left|s_{k_{1}}-s_{k_{2}}\right|}^{2}$ for a pair ($s_{k_{1}},s_{k_{2}}$), where V and E are the sets of vertices and edges, respectively. Given a connected, weighted graph, an MST is an acyclic subgraph that connects all vertices and minimizes the sum of the weights of the included edges. Because of this property, MST is routinely used to develop transportation and telecommunication networks [198]. For a regular grid, MST is not unique, as the inter-point distances are not distinct. However, a unique random MST can be curated by simply adding small random values to the distances [187, 160].

### 4.2 Spatial modeling

#### 4.2.1 Spatial fused lasso

In a recent study [157] of the temperature-salinity relationship in the Atlantic Ocean, Li et al. (2019) consider Eq. 1 and elegantly promote spatial homogeneity of the coefficients by considering fused lasso penalties [151, 199]: $|\beta_{0}^{mm^{\prime }}\left(s_{k_{1}}\right)-\beta_{0}^{mm^{\prime }}\left(s_{k_{2}}\right)|\approx 0$ and $|\beta_{1}^{mm^{\prime }}\left(s_{k_{1}}\right)-\beta_{1}^{mm^{\prime }}\left(s_{k_{2}}\right)|\approx 0$ for $\left(s_{k_{1}},s_{k_{2}}\right)\in E$, in a frequentist setup. These constraints are intuitive, as it is reasonable to expect both the degree of co-expression, $\beta_{1}^{mm^{\prime }}(s)$, and the effect of “unmeasured” factors, $\beta_{0}^{mm^{\prime }}(s)$, to remain homogeneous across adjacent or connected locations. Extending this idea, a Bayesian fused lasso [200, 153] approach can be considered with Laplacian priors on the pair-wise differences of the coefficients as

(3) $$\begin{array}{l} \pi (\beta_{0}^{mm^{\prime }}|\ldots )\propto \prod_{(s_{k_{1}},s_{k_{2}})\in E}\;\;exp\left(-\frac{\lambda_{0}}{\sigma }|\beta_{0}^{mm^{\prime }}\left(s_{k_{1}}\right)-\beta_{0}^{mm^{\prime }}\left(s_{k_{2}}\right)|\right),\beta_{0}^{mm^{\prime }}={\left(\beta_{0}^{mm^{\prime }}\left(s_{1}\right),\ldots ,\beta_{0}^{mm^{\prime }}\left(s_{n}\right)\right)}^{T},\\ \pi (\beta_{1}^{mm^{\prime }}|\ldots )\propto \prod_{(s_{k_{1}},s_{k_{2}})\in E}\;\;exp\left(-\frac{\lambda_{1}}{\sigma }|\beta_{1}^{mm^{\prime }}\left(s_{k_{1}}\right)-\beta_{1}^{mm^{\prime }}\left(s_{k_{2}}\right)|\right),\beta_{1}^{mm^{\prime }}={\left(\beta_{1}^{mm^{\prime }}\left(s_{1}\right),\ldots ,\beta_{1}^{mm^{\prime }}\left(s_{n}\right)\right)}^{T}, \end{array}$$

where $\sigma^{2}$ is the variance of the error term $\epsilon \left(s_{k}\right),\;{\;\lambda }_{0}$ and $\lambda_{1}$ are regularization parameters that control the strength of fusion and are assumed to follow gamma priors. Note that $\sigma^{2}$ is only present in the Gaussian model (Eq. 1) and could be omitted from the above exponents for simplicity. We discuss the resemblance of the prior to the intrinsic CAR (ICAR) prior [146] and, more generally, the intrinsic GMRF (IGMRF) prior [150] in the Supplementary Material. Theoretically, using the $L_{1}$ distance seems appealing, as it has the potential to achieve better spatial smoothing by “exactly” fusing coefficient values at adjacent locations, unlike the $L_{2}$ distance implied by the ICAR prior. This is analogous to how lasso regression enforces sparsity in solutions, while ridge regression only shrinks effect sizes toward 0 [201]. For transparency, such a spatial fused lasso prior has already been proposed in the existing literature [192, 202].

#### 4.2.2 Spatial fused horseshoe

In variable selection problems, failure of the Bayesian lasso or Laplacian prior to achieve exact sparsity, unlike the frequentist analog, has been reported, while also underestimating larger effect sizes [203, 204, 205]. Consequently, the Bayesian fused lasso might struggle to promote spatial smoothness and preserve distinct local features simultaneously. For variable selection, the advantages of the horseshoe prior have been convincingly demonstrated to handle unknown sparsity and large outlying signals [154, 206, 207]. The horseshoe prior belongs to the class of global-local shrinkage priors [208], characterized by a “global” hyperparameter that controls overall shrinkage, while “local” hyperparameters control shrinkage per coefficient. Following recent developments on Bayesian fused horseshoe approach [156, 209], we assume that

(4) $$\begin{array}{l} \beta_{0}^{mm^{\prime }}(s_{k_{i}^{1}})-\beta_{0}^{mm^{\prime }}(s_{k_{i}^{2}})|\Lambda_{0i}^{2},\tau_{0}^{2},\sigma^{2}\sim N\left(0,\Lambda_{0i}^{2}\tau_{0}^{2}\sigma^{2}\right),\;\Lambda_{0i}\sim C^{+}(0,\;1),\;\tau_{0}\sim C^{+}(0,\;1)\\ \beta_{1}^{mm^{\prime }}(s_{k_{i}^{1}})-\beta_{1}^{mm^{\prime }}(s_{k_{i}^{2}})|\Lambda_{1i}^{2},\tau_{1}^{2},\sigma^{2}\sim N\left(0,\Lambda_{1i}^{2}\tau_{1}^{2}\sigma^{2}\right),\;\Lambda_{1i}\sim C^{+}(0,\;1),\;\tau_{1}\sim C^{+}(0,\;1) \end{array}$$

independently, where ($s_{k_{i}^{1}},s_{k_{i}^{2}}$) are the nodes or locations associated with the i-th edge from $E.\;\tau_{0}$ and $\tau_{1}$ are the global hyper-parameters, $\Lambda_{0i}$’s and $\Lambda_{1i}$’s are the local hyperparameters, and $C^{+}$ stands for the half-Cauchy distribution. The resulting conditional prior PDFs of ($\beta_{0}^{mm^{\prime }},\beta_{1}^{mm^{\prime }}$) are

(5) $$\pi (\beta_{0}^{mm^{\prime }}|.)\;\propto \prod_{i=1}^{p}\;\;\frac{1}{\Lambda_{0i}\tau_{0}\sigma }\;exp\left[\;-\;\frac{{\left(\beta_{0}^{mm^{\prime }}(s_{k_{i}^{1}})\;-\;\beta_{0}^{mm^{\prime }}(s_{k_{i}^{2}})\right)}^{2}}{2\Lambda_{0i}^{2}\tau_{0}^{2}\sigma^{2}}\right]\;\pi (\beta_{1}^{mm^{\prime }}|.)\;\propto \prod_{i=1}^{p}\;\;\frac{1}{\Lambda_{1i}\tau_{1}\sigma }\;exp\left[-\frac{{\left(\beta_{1}^{mm^{\prime }}(s_{k_{i}^{1}})\;-\;\beta_{1}^{mm^{\prime }}(s_{k_{i}^{2}})\right)}^{2}}{2\Lambda_{1i}^{2}\tau_{1}^{2}\sigma^{2}}\right]$$

The spatial fused horseshoe prior, with its half-Cauchy priors on both global and local hyperparameters, has heavy tails that allow substantial differences between neighboring locations to escape excessive shrinkage, unlike the Laplacian prior. At the same time, its infinitely tall spike at zero strongly shrinks small differences toward zero. This dual behavior enables the prior to preserve spatial homogeneity while still accommodating sharp local variations. The relation of such a locally adaptive fusion prior to the general class of GMRF priors was first highlighted by Faulkner and Mining (2017) [210] in a longitudinal modeling context, encouraging fusion between coefficients across time points. In the Supplementary Material, we provide a discussion on this relation and details of the Gibbs sampling steps, which include the Pólya-Gamma data augmentation strategy [193, 211] for the NB model (Eq. 2).

One critical aspect that deserves elucidation is the assumption of independence between edge-wise differences. Specifically, for any two edges i, $i^{\prime }$, $\beta_{1}^{mm^{\prime }}(s_{k_{i}^{1}})-\beta_{1}^{mm^{\prime }}(s_{k_{i}^{2}})$ and $\beta_{1}^{mm^{\prime }}(s_{k_{i^{\prime }}^{1}})-\beta_{1}^{mm^{\prime }}(s_{k_{i^{\prime }}^{2}})$ are assumed to be independent in Eq. 4, conditional on the hyperparameters. To see how this assumption could be problematic for a general graph (not the MST), we briefly highlight one example from Rue and Held (2005) [150] provided in the context of IGMRF priors. Suppose there are only three locations A,B, and C, all neighbors of each other. Letting $e_{1}=\beta_{1}^{mm^{\prime }}\left(A\right)-\beta_{1}^{mm^{\prime }}\left(B\right),\;e_{2}=\beta_{1}^{mm^{\prime }}(B)-\beta_{1}^{mm^{\prime }}(C)$, and $e_{3}=\beta_{1}^{mm^{\prime }}(C)-\beta_{1}^{mm^{\prime }}(A)$, Eq. 4 proceeds to assume $e_{1}$, $e_{2}$, $e_{3}$ are independent and normally distributed with non-identical parameters. However, we notice that there is a “hidden” linear constraint, $e_{1}+e_{2}+e_{3}=0$, that directly contradicts the model assumption of independence. Analogously, using a highly connected or dense spatial neighborhood graph G introduces numerous hidden constraints corresponding to the cycles in G. Interestingly enough, as demonstrated in Theorem 1 of the Supplementary Material, the hidden constraints do not require to be explicitly accounted for since the posterior sampling distribution of $\beta_{0}$ and $\beta_{1}$ (based on the priors in Eq. 5) remains unaffected. However, penalizing too many edge-wise differences and the implicit dependency might lead to over-smoothing and loss of local structures. Thus, the use of MST becomes crucial, as it is acyclic (no hidden constraints) and eliminates redundant relationships. Additionally, a sparser G enables faster Cholesky decomposition of the precision matrix, enhancing computational efficiency.

### 4.3 Hypothesis testing

We consider two types of hypothesis tests: 1) global test: to determine the significance of average association across the entire tissue domain $\left(H_{0}\;:\bar{\beta_{1}^{mm^{\prime }}}=\frac{1}{n}\sum_{k=1}^{n}\;\beta_{1}^{mm^{\prime }}\left(s_{k}\right)=0\right)$, based on the credible interval [212] of $\bar{\beta_{1}^{mm^{\prime }}}$, and 2) local test: to determine the significance of location-level association $\left(H_{0}^{k}\;:\beta_{1}^{mm^{\prime }}\left(s_{k}\right)=0\right)$, directly based on the credible intervals of $\beta_{1}^{mm^{\prime }}\left(s_{k}\right)$’s. Additionally, in the genomic context, having a measure analogous to the frequentist p-value is often beneficial. To this end, we utilize a metric termed the probability of direction $\left(p_{d}\right)$, which quantifies the probability (between 0.5 and 1 ) that a parameter has an effect in a specific direction, either positive or negative [213, 214. Mathematically, it is defined as the proportion of the posterior distribution that shares the same sign as the median. $p_{d}$ resembles a two-sided frequentist p-value as $p_{\text{two-sided}}=2\left(1-p_{d}\right)$. It is implemented in the R package bayestestR [213].

### 4.4 Simulation design

We consider two different simulation designs as outlined below, for assessing the type 1 error and power of the model proposed in Eq. 2. The locations at which the variables are simulated are the same as the previously discussed cutaneous melanoma dataset (n=293). We have observed that the results remain unaffected when a randomly generated set of locations or other real data-based sets of locations are used.

#### 4.4.1 Simulation design 1

In the first design, we directly consider the model from Eq. 2 to generate ($X^{m},X^{m^{\prime }}$) based on two steps. First, we generate an NB-distributed RV, $X^{m^{\prime }}$, using Gaussian copula [127], incorporating spatial dependency between the observations via a kernel covariance matrix H with an exponential kernel on the $L_{2}$ distances and varying lengthscale (l) parameters [114]. Then, based on the simulated $X^{m^{\prime }}$, we generate $X^{m}$ following Eq. 2 with a fixed slope $\beta_{1}^{mm^{\prime }}\left(s_{k}\right)=\nu$. More specifically, for a fixed choice of l, failure probability $\psi_{m^{\prime }}$, dispersion parameter $r_{m^{\prime }}$ for variable $m^{\prime }$, and dispersion parameter $r_{m}$ for variable m, we consider the following steps

1. Simulate a spatially autocorrelated normal RV of size n using a Gaussian process (GP) model:
$$Z^{m^{\prime }}\sim MVN\;(0,H),\;H_{k_{1}k_{2}}=exp\left(-\frac{\left|s_{k_{1}}\;-\;s_{k_{2}}\right|}{l}\right),$$
2. Transform to a vector of uniform RVs using the standard normal CDF (Φ):
$$U^{m^{\prime }}=\Phi (Z^{m^{\prime }}),$$
3. Convert to a vector of NB RVs using the inverse CDF of $NB\left(\psi_{m^{\prime }},r_{m^{\prime }}\right)$, denoted by $F_{NB\left(\psi_{m^{\prime }},r_{m^{\prime }}\right)}^{-1}$:
$$X^{m^{\prime }}=F_{NB\left(\psi_{m^{\prime }},r_{m^{\prime }}\right)}^{-1}(U^{m^{\prime }}),$$
  Each element of the resulting vector $X^{m^{\prime }}$ retains the marginal NB distribution, $NB\left(\psi_{m^{\prime }},r_{m^{\prime }}\right)$, where $\psi_{m^{\prime }}$ is the failure probability and $r_{m^{\prime }}$ is the dispersion.
4. Generate the link function to simulate variable m with a fixed slope of $\beta_{1}^{mm^{\prime }}\left(s_{k}\right)=\nu$ (2):
$$\eta_{m}=\beta_{0}^{mm^{\prime }}+\nu \;log(X^{m^{\prime }}+1),\;\beta_{0}^{mm^{\prime }}\sim MVN(0,\;0.5H),$$
5. Convert the link vector to failure probabilities $\psi_{m}$ and simulate $X^{m}$ from the NB distribution:
$$\psi_{m}=\frac{exp\left(\eta_{m}\right)}{1\;+\;exp\left(\eta_{m}\right)},\;X^{m}\sim NB\left(\psi_{m},r_{m}\right)$$
  where $r_{m}$ is a prefixed dispersion parameter. The k-th element of $X^{m}$ follows $NB\left(\psi_{mk},r_{m}\right)$, where $\psi_{m}={\left(\psi_{m1},\ldots ,\psi_{mn}\right)}^{T}$ and $\eta_{m}={\left(\eta_{m1},\ldots ,\eta_{mn}\right)}^{T}$.

Three values of the lengthscale l are considered, l=3.6,7.2,18, with the corresponding structure of H displayed in Fig. 4A. The failure probability of variable $m^{\prime }$ and dispersion parameters are kept fixed, $\psi_{m^{\prime }}=0.5$, $r_{m}=r_{m^{\prime }}=1$. The slope parameter ν is varied between {−0.75, −0.5, −0.25, 0, 0.25, 0.5, 0.75}, with negative and positive values representing negative and positive association, respectively. Higher absolute value of ν dictates the strength of association, and ν=0 corresponds to the null model, i.e., $X^{m}$ and $X^{m^{\prime }}$ are independent.

#### 4.4.2 Simulation design 2

In this design, ($X^{m},X^{m^{\prime }}$) are simulated jointly using a bivariate Gaussian copula and spatial dependency incorporated using a bivariate GP framework, where the joint covariance matrix has a Kronecker product structure, comprising a 2 × 2 correlation matrix and the distance kernel covariance matrix H (Eq. 9.11 from Banerjee et al. (2014)[97] and Eq. 6 from the Supplementary Material). Specifically, we consider the following steps

1. Simulate spatially cross-correlated normal RVs:
$${\left(Z^{m},Z^{m^{\prime }}\right)}^{T}\sim MVN\left(\left[\begin{array}{l} 0\\ 0 \end{array}\right],\Sigma =\left[\begin{array}{ll} 1 & \nu \\ \nu & 1 \end{array}\right]⊗H\right),H_{k_{1}k_{2}}=exp\left(-\frac{\left|s_{k_{1}}\;-\;s_{k_{2}}\right|}{l}\right),$$
2. Transform to uniform RVs using the standard normal CDF (Φ):
$$U^{m}=\Phi \left(Z^{m}\right),\;U^{m^{\prime }}=\Phi (Z^{m^{\prime }}),$$
3. Convert to NB random variables using the inverse CDFs:
$$X^{m}=F_{NB\left(\psi_{m},r_{m}\right)}^{-1}\left(U^{m}\right),\;X^{m^{\prime }}=F_{NB\left(\psi_{m^{\prime }},r_{m^{\prime }}\right)}^{-1}(U^{m^{\prime }}),$$
  Each element of the resulting vectors $X^{m}$ and $X^{m^{\prime }}$ retain the marginal NB distributions, $NB\left(\psi_{m},r_{m}\right)$ and $NB\left(\psi_{m^{\prime }},r_{m^{\prime }}\right)$, respectively, where $\psi_{m}$, $\psi_{m^{\prime }}$ are failure probabilities and $r_{m}$, $r_{m^{\prime }}$ are dispersions.

The lengthscale l is varied between {0.6, 1.8, 3.6, 7.2}. The failure probabilities and dispersion parameters are kept fixed, $\psi_{m}=\psi_{m^{\prime }}=0.5$, $r_{m}=r_{m^{\prime }}=1$. The parameter ν is varied between {−0.75, −0.5, −0.25, 0, 0.25, 0.5, 0.75}, with similar implications on the direction and strength of association as before.

### 4.5 Competing methods

We compare SpaceBF to five different methods: 1) MERINGUE [98] with the Delaunay triangulation network, 2) SpatialDM [102] with the kernel weight matrix (lengthscale 1.2 as used in the original paper for the melanoma dataset), 3) Lee’s L [110] with k-NN network (implemented in R package spdep [215], k=1), 4) SpaGene [100], and 5) PearsonCorr, which is the simple Pearson correlation. We do not use the original packages of LIANA+ [104] and Voyager [117], as the former essentially implements SpatialDM and the latter is a direct application of Lee’s L. Table 1 summarizes the methods in terms of their assumptions and limitations. We also attempted to evaluate the performance of SpatialCorr [176] and Copulacci [105]. SpatialCorr was straightforward to use, but it proved highly sensitive to the choice of the lengthscale l in its innovative use of the spatial covariance matrix H. Copulacci was slightly difficult to use and will be benchmarked in a future study.

## Supplementary Material

Supplement 1

## Figures and Tables

**Figure 5:**
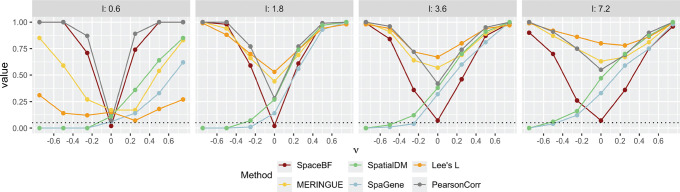
Comparison of global tests under simulation design 2 for lengthscale l between {0.6, 1.8, 3.6, 7.2}.

**Table 1:** Comparison of the methods in terms of the underlying assumptions.

Method	Central metric or concept	Global and local co-expression tests	Test type	Potential sensitivity

**SpaceBF**	Spatially varying coefficients model with NB distribution	Global and local	Exact	MST-based spatial network
MERINGUE	Bivariate Moran’s *I*	Global	Permutation test	Neighborhood network
SpatialDM	Bivariate Moran’s *I*	Global and local	Permutation or exact test	Lengthscale in the kernel weight matrix
LIANA+	Bivariate Moran’s *I* and cosine similarity	Global and local	Permutation test	As above
Voyager	Lee’s *L*	Global and local	Permutation test	Neighborhood network
SpaGene	k-NN network and earth mover’s distance	Global but with local interaction scores	Permutation test	Choice of *k*
PearsonCorr	Pearson correlation	Global	Permutation or	None
SpatialCorr *	Spatial kernel-weighted sample correlation	Global and local	Permutation test	Lengthscale in the kernel weight matrix
Copulacci *	Bivariate Poisson distribution along a neighborhood network	Global but with local interaction scores	Permutation test	Neighborhood network and fixed correlation term across locations

* methods that are not evaluated in the simulations.

## Data Availability

The datasets analyzed are publicly available from the links provided in the corresponding manuscripts: 1) cutaneous melanoma [39] and 2) cSCC [161]. The spatial proteomics dataset is available from the corresponding author upon a reasonable request. A GitHub R package, termed SpaceBF, implementing the proposed method and the datasets in “.RData” format is available at https://github.com/sealx017/SpaceBF/.
